# A Systematic Investigation of Accuracy and Response Time Based Measures Used to Index ANS Acuity

**DOI:** 10.1371/journal.pone.0163076

**Published:** 2016-09-16

**Authors:** Julia Felicitas Dietrich, Stefan Huber, Elise Klein, Klaus Willmes, Silvia Pixner, Korbinian Moeller

**Affiliations:** 1 Leibniz-Institut fuer Wissensmedien, Tuebingen, Germany; 2 Department of Psychology, Eberhard Karls University, Tuebingen, Germany; 3 Department of Neurology, Section Neuropsychology, University Hospital, RWTH Aachen, Aachen, Germany; 4 Institute of Applied Psychology, UMIT–The Health and Life Sciences University, Hall in Tyrol, Austria; 5 LEAD Graduate School, Eberhard Karls University, Tuebingen, Germany; Katholieke Universiteit Leuven, BELGIUM

## Abstract

The approximate number system (ANS) was proposed to be a building block for later mathematical abilities. Several measures have been used interchangeably to assess ANS acuity. Some of these measures were based on accuracy data, whereas others relied on response time (RT) data or combined accuracy and RT data. Previous studies challenged the view that all these measures can be used interchangeably, because low correlations between some of the measures had been observed. These low correlations might be due to poor reliability of some of the measures, since the majority of these measures are mathematically related. Here we systematically investigated the relationship between common ANS measures while avoiding the potential confound of poor reliability. Our first experiment revealed high correlations between all accuracy based measures supporting the assumption that all of them can be used interchangeably. In contrast, not all RT based measures were highly correlated. Additionally, our results revealed a speed-accuracy trade-off. Thus, accuracy and RT based measures provided conflicting conclusions regarding ANS acuity. Therefore, we investigated in two further experiments which type of measure (accuracy or RT) is more informative about the underlying ANS acuity, depending on participants’ preferences for accuracy or speed. To this end, we manipulated participants’ preferences for accuracy or speed both explicitly using different task instructions and implicitly varying presentation duration. Accuracy based measures were more informative about the underlying ANS acuity than RT based measures. Moreover, the influence of the underlying representations on accuracy data was more pronounced when participants preferred accuracy over speed after the accuracy instruction as well as for long or unlimited presentation durations. Implications regarding the diffusion model as a theoretical framework of dot comparison as well as regarding the relationship between ANS acuity and math performance are discussed.

## Introduction

A prominent theory in numerical cognition postulates that the origins of our symbolic math abilities are rooted in a nonverbal, evolutionary old system for approximately representing non-symbolic numerical quantities, which we share with many nonhuman species [1–11]. This system, often referred to as either number sense, analogue magnitude system, or approximate number system (ANS) [5,6,8,9,12–14], is assumed to support rapid comparison or estimation of numerosities as well as basic arithmetic operations with non-symbolic quantities [1,4].

On the neural level, numerous studies investigated the underlying mechanisms of this system (see [10,15] for reviews). For instance, using single-cell recordings, Nieder and colleagues found numerosity-selective neurons in lateral prefrontal and posterior parietal cortex of monkeys [16–18]. These neurons showed maximum firing rate for one specific numerosity (i.e., their “preferred numerosity”). Moreover, their neural activation decreased gradually the more a presented numerosity deviated from the preferred numerosity [16,18]. Thus, the activity of all numerosity-selective neurons followed a series of numerically overlapping tuning curves with peak activation at the preferred numerosity, which represent the respective numerosities [13,19]. The overlap of tuning curves increased with numerosity [10,14,16]. There is evidence from a series of human brain imaging studies suggesting that such numerosity-selective neurons also exist in humans [20–22]. For example, using a representational similarity approach, Lyons et al. (2015) demonstrated that numerosities are represented by overlapping tuning curves, which show an increasing overlap for larger numerosities [20].

Currently, two models describe the representation of non-symbolic quantities with increasingly overlapping tuning curves: on the one hand, the *linear model* (or *scalar variability model*) postulates equally spaced tuning curves (i.e., a linear scaling) with increasing variability (i.e., width of the curves, [11,23,24]). On the other hand, the *logarithmic model* (or *log-Gaussian model*) proposes tuning curves with fixed variability on a logarithmic scale ([25–27], see [14,28] for reviews). Both models make highly similar predictions at the behavioral level [14,28]. However, at the neural level, single-cell recording studies with monkeys provided evidence for the logarithmic model [28,29].

The overlapping tuning curves appear to influence behavior in tasks, which revert to these representations, e.g., the non-symbolic dot comparison task, because studies repeatedly yielded behavioral effects such as the ratio or distance effect [27,30–34]. These effects are assumed to result from the different degree of overlap between the tuning curves. The overlap of the tuning curves is related to the ratio between the to-be-compared numerosities and affects discrimination performance [5,12,16,18]: The larger the overlap between the tuning curves, the worse is discrimination performance. This pattern of overlap explains the *numerical ratio effect* (NRE): numerosities with a larger ratio (e.g., 9 vs. 10 dots; ratio 9:10 = 0.9) are more difficult to discriminate than numerosities with a smaller ratio (e.g., 5 vs. 10 dots; ratio 5:10 = 0.5). Tuning curves for numerosities with a large ratio overlap to a higher degree than tuning curves for numerosities with a small ratio. Similarly, the varying degree of overlap also explains the *numerical distance effect* (NDE): less distant numerosities (e.g., 9 vs. 10 dots) are harder to discriminate than more distant numerosities (e.g., 5 vs. 10 dots; [27]). Tuning curves for less distant numerosities overlap to a higher degree than for more distant numerosities, just like for the ratio effect. In turn, this overlap results in reduced discrimination performance for less distant numerosities [12,13,29,35]. As the NRE and the NDE are both thought to reflect the underlying representations of non-symbolic numerosities (subsequently referred to as ANS representations), these behavioral effects have often been employed to assess ANS representations [2,30,31,36–44].

However, beside these measures there are several other measures, which have been used to assess the acuity of the underlying ANS representations (subsequently referred to as ANS acuity; see [45–47] for reviews or [48,49] for meta-analyses), implicitly assuming that all these measures reflect ANS acuity to a similar degree [40]. All these measures are computed based on the results of a non-symbolic dot comparison task, which is the standard paradigm to assess ANS acuity in children and adults [45,46]. In this task, participants are asked to compare two dot sets and select the set containing more dots. Some of these measures are based on accuracy data whereas other measures are based on response times (RT) or consider combinations of both, accuracy and RT [45,46].

In the following, we will first describe accuracy based measures and how they are expected to be interrelated before introducing RT based measures of ANS acuity and their expected interrelations. This systematic overview will allow for a first evaluation of the assumption that all these measures reflect ANS acuity similarly and, thus, may be used interchangeably. Subsequently, we will also evaluate this assumption empirically in three experiments.

### Accuracy based measures

Besides mean accuracy, accuracy based measures include the Weber fraction, the numerical distance effect (NDE_acc), and the numerical ratio effect (NRE_acc) calculated from accuracy data. All these measures can be derived from the theoretical notion that the ANS underlies our ability to compare numerosities and represents these numerosities by overlapping Gaussian tuning curves [14]. When the ANS indeed underlies our ability to compare numerosities [1,4], ANS acuity should affect performance in non-symbolic dot comparison: more accurate ANS representations should result in better discrimination performance and, thus, a larger percentage of correctly answered trails in individual participants leading to higher mean accuracy.

Moreover, both the NDE_acc and the NRE_acc have been used to index ANS acuity [30,31,39,40]. The accuracy distance effect (ratio effect) describes the finding that error rates increase as the numerical distance (ratio; i.e., smaller numerosity divided by larger numerosity) between two to-be-compared numbers decreases (increases) [27]. NDE_acc and NRE_acc are calculated using a generalized linear model with numerical distance (for NDE_acc) or the ratio (for NRE_acc) between the to-be-compared numerosities as predictor and the binary response (correct response vs. error) as dependent variable. The resulting slope for distance/ ratio reflects the NDE_acc/ NRE_acc. Both effects are assumed to reflect consequences of the overlapping Gaussian tuning curves. The larger the overlap between the ANS representations, the harder becomes the discrimination between to-be-compared numerosities. Thus, the smaller the distance between two to-be-compared numerosities or the larger their ratio, the larger is the overlap between the respective ANS representations, and, hence, the worse becomes discrimination performance. It has been assumed that the more precise (i.e., the less overlapping) ANS representations are, the smaller the distance and the ratio effect (e.g., [45]). Thus, for participants with a more accurate ANS the decrease in accuracy with increasing task difficulty should be less pronounced (see [46] for a discussion of misinterpretations in case of floor effects).

Furthermore, the ANS is assumed to follow Weber’s Law, because the discrimination of two numerosities depends on their ratio [26,50] (but see [40]). Hence, the Weber fraction is often used as a measure of ANS acuity in the individual participant [51–53]. The Weber fraction is a direct measure of ANS acuity, because it directly reflects the width of the Gaussian tuning curves (i.e., ANS representations [54]): the smaller the Weber fraction, the narrower and the less overlapping are the Gaussian tuning curves and, thus, the higher is ANS acuity. For the linear model assuming linear scaling of numerosities represented by the overlapping tuning curves, the Weber fraction is estimated from accuracy data by

$$p_{\text{correct}}(r,w)=1-\frac{1}{2}erfc\;\left(\frac{\left|r-1\right|}{\sqrt{2}w\sqrt{r^{2}+1}}\right)$$ (1)

(with *r* being the ratio between the larger and the smaller numerosity and erfc being the complementary Gaussian error function [54]).

On the other hand, for the logarithmic model suggesting logarithmic scaling, the Weber fraction is estimated from the probability of choosing the right numerosity by the following formula:

$$p_{choose\;right}(r_{num},w)=\frac{1}{2}+\frac{1}{2}erf\;\left(\frac{{\text{log}}_{2}(r_{num})}{\sqrt{2}(\sqrt{2}w)}\right)$$ (2)

(with *r*_*num*_ being the ratio between the right and the left numerosity and erf being the error function of the normal distribution, [21,55]). This corresponds to:

$$p_{correct}(r_{num},w)=1-\frac{1}{2}erfc\;\left(\frac{\left|{\text{log}}_{2}(r_{num})\right|}{\sqrt{2}(\sqrt{2}w)}\right)$$ (3)

Only recently, a modification of this logarithmic model has been proposed, which accounts for the effect of visual properties of the stimuli on performance in a non-symbolic dot comparison task [55]. Previous studies demonstrated that task performance as well as the Weber fraction were strongly influenced by visual properties of the stimuli [56–58]. This novel model allows for separating the effects of ANS acuity from effects of non-numeric visual properties by fitting a generalized linear model to choice data with the intercept *β*_*side*_ and predictors for the logarithm of the ratio of numerosity (*r*_*num*_), size (log_2_(*r*_*size*_), i.e., the sum of the logarithms of item surface area and total surface area), and spacing (log_2_(*r*_*spacing*_), i.e., the sum of the logarithms of field area and sparsity) of the stimuli on the right and the left side (for a detailed description of the model see [55]):

$$p_{choose\;right}=\frac{1}{2}+\frac{1}{2}erf\;\left(\frac{\beta_{side}+\beta_{num}{\text{log}}_{2}(r_{num})+\beta_{size}{\text{log}}_{2}(r_{size})+\beta_{spacing}{\text{log}}_{2}(r_{spacing})}{\sqrt{2}}\right)$$ (4)

Based on this model, the Weber fraction w is $w=\frac{1}{\sqrt{2}\beta_{num}}$ [55].

### Interrelations between accuracy based measures

All these accuracy based measures are derived theoretically and are assumed to assess the same underlying ANS representations [40,45,46,48,49]. Moreover, these measures are also mathematically related. We demonstrate this aspect by focusing on the relationship between the numerical parameters (e.g., distance and ratio), which determines the relationship between the resulting measures (e.g., for the distance effect and the ratio effect), because they are all determined using a generalized linear mixed model. First, distance and ratio are inversely related, when calculating the ratio by dividing the smaller numerosity by the larger numerosity [i.e., R = min(n_1_, n_2_)/max(n_1_, n_2_)] and defining distance as D = |n_1_ –n_2_|. With decreasing distance between to-be-compared numerosities (towards a minimum of 1), their ratio increases towards 1, getting closer to 1 with increasing numerosity. However, this does not apply for all combinations of two numerosities (e.g., comparing 1 and 2 dots results in a distance of 1, but the ratio is 0.5, clearly smaller than 1). However, it holds for the majority of possible combinations of two numbers. For the item set used in the current experiments, distance and ratio of the to-be-compared numerosities were negatively correlated (Pearson product-moment correlation coefficient: *r* = -.898 and Spearman rank correlation coefficient: *r*_*s*_ = -.911). The NDE_acc as well as the NRE_acc were calculated using a generalized linear model with accuracy as binary dependent variable and distance or ratio as predictor. As the predictor variables distance and ratio were negatively correlated, the resulting slopes for distance and ratio (i.e., the NDE_acc and the NRE_acc) were inversely related as well.

Second, regarding the relationship between Weber fraction and NRE_acc, the formula of the linear (model 1) above can be simplified as follows:

$$p_{\text{correct}}(r,w)=1-\frac{1}{2}erfc\;\left(\frac{\left|r-1\right|}{\sqrt{2}w\sqrt{r^{2}+1}}\right)=\Phi (\frac{\left|r-1\right|}{w\sqrt{r^{2}+1}}),$$

because

$$\Phi (x)=1-\frac{1}{2}erfc\;\left(\frac{x}{\sqrt{2}}\right)$$ (5)

(with *r* being the ratio between the larger and the smaller numerosity, erfc being the complementary Gaussian error function and Φ being the cumulative normal distribution function).

Replacing 1/*w* by a parameter *β*_*linear*_, a probit model results with the predictor variable $\frac{\left|r-1\right|}{\sqrt{r^{2}+1}}$. Thereby, when estimating a Weber fraction, *β*_*linear*_ (or 1/*w*) is effectively fitted against this predictor variable for each participant individually. Thus, to obtain an estimate of the correlation between Weber fraction and ratio R in a sample of participants, the inverse of the predictor has to be calculated, i.e., $\frac{\sqrt{r^{2}+1}}{\left|r-1\right|}$. For the items used in the present experiments, the Weber fraction estimates correlated at *r* = .841 and *r*_*s*_ = 1 with the ratio R. The opposite pattern resulted when correlating the Weber fraction estimates with the distance D (for our item set: *r* = -.739, *r*_*s*_ = -.911), because distance and ratio are inversely related.

A highly similar correlational pattern of the Weber fraction with either NDE_acc or NRE_acc is also expected under the logarithmic model. The (formula 3) above can be simplified as follows:

$$p_{correct}(r_{num},w)=1-\frac{1}{2}erfc\;\left(\frac{\left|{\text{log}}_{2}(r_{num})\right|}{\sqrt{2}(\sqrt{2}w)}\right)=\Phi \left(\frac{\left|{\text{log}}_{2}(r_{num})\right|}{\sqrt{2}w}\right),$$

because

$$\Phi (x)=1-\frac{1}{2}erfc\;\left(\frac{x}{\sqrt{2}}\right)$$ (6)

with *r*_*num*_ being the ratio of the right numerosity to the left numerosity, erfc being the complementary Gaussian error function, and Φ being the cumulative normal distribution function [21,55]. Again, replacing $1/(w\sqrt{2})$ with a parameter *β*_log_, results in a probit model with the predictor |log_2_(*r*_*num*_)|. Thereby, when computing the Weber fraction, *β*_log_ (or $1/(w\sqrt{2})$) is effectively fitted against this predictor variable. Thus, to get an estimate for the correlation of the Weber fraction with the predictors, ratio R or distance D, $\frac{\sqrt{2}}{\left|{\text{log}}_{2}(r)\right|}$ has to be calculated. Using this predictor, the correlation between the Weber fraction (estimated from the logarithmic model) and the ratio R as well as the distance D can be estimated. For the items used in the present experiments, the Weber fraction estimates correlated at *r* = .845 and *r*_*s*_ = 1 with the ratio R. Again, the opposite pattern resulted for the correlation between the Weber fraction estimates of the logarithmic model and the distance D (for our item set: *r* = -.742, *r*_*s*_ = -.911), because distance and ratio are inversely related. Obviously, both Weber predictor estimates (from the linear and the logarithmic model) are highly correlated: *r* = 1, *r*_*s*_ = 1.

A recently proposed modification supplemented the logarithmic model by including the intercept *β*_*side*_ and additional predictors for the logarithm of the ratio of size and spacing [55]:

$$p_{choose\;right}(r,w)=1-\frac{1}{2}erfc\;\left(\frac{\beta_{side}+\beta_{num}{\text{log}}_{2}(r_{num})+\beta_{size}{\text{log}}_{2}(r_{size})+\beta_{spacing}{\text{log}}_{2}(r_{spacing})}{\sqrt{2}}\right)$$

$$=\Phi \left(\beta_{side}+\beta_{num}{\text{log}}_{2}(r_{num})+\beta_{size}{\text{log}}_{2}(r_{size})+\beta_{spacing}{\text{log}}_{2}(r_{spacing})\right)$$ (7)

with β_side_ being the intercept, *r*_*num*_ being the ratio of the right numerosity to the left numerosity, *r*_*size*_ being the ratio of the right (dot) size to the left (dot) size, *r*_*spacing*_ being the ratio of the right spacing of the dots to the left spacing, erfc being the complementary Gaussian error function, and Φ being the cumulative normal distribution function [21,55]. Therefore, the correlations with the NDE_acc and the NRE_acc should be similar for this modified logarithmic model when compared with the original logarithmic model.

**Fig 1:**
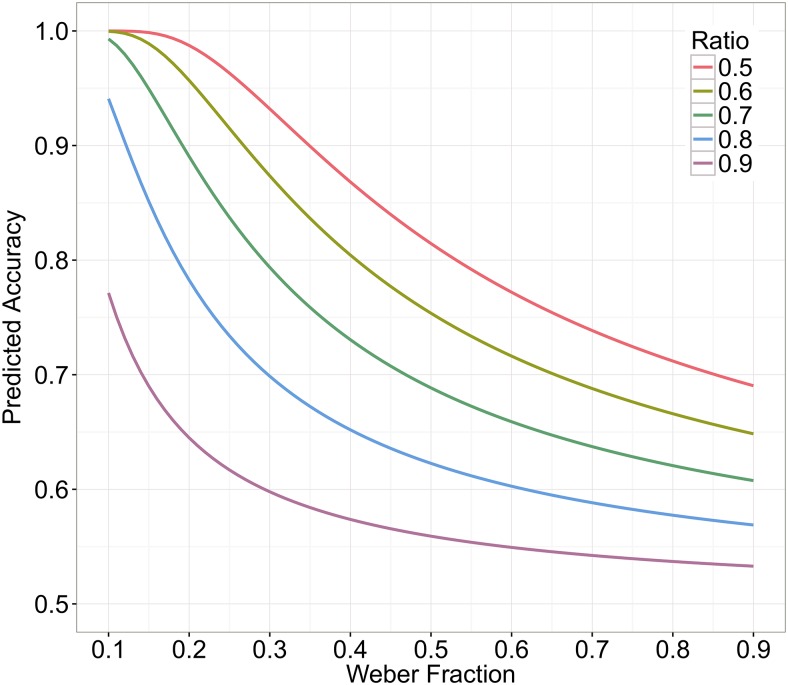
Interrelations of predicted accuracy and Weber fraction (calculated from the linear model) for given ratios.

Regarding the relationship between Weber fraction and mean accuracy, it can be shown that they are negatively related. To do so, we calculated predicted accuracies based on Eq 1 for given Weber fractions and ratios. Fig 1 shows the relationship between predicted accuracy and Weber fractions ranging from 0.1 to 0.9 for five different ratios (ranging from 0.5 to 0.9). As can be seen in this figure, there is always a negative relationship between predicted accuracy and Weber fraction irrespective of ratio (i.e., a monotonously decreasing pattern, and thus, *r*_*s*_ = -1).

**Fig 2:**
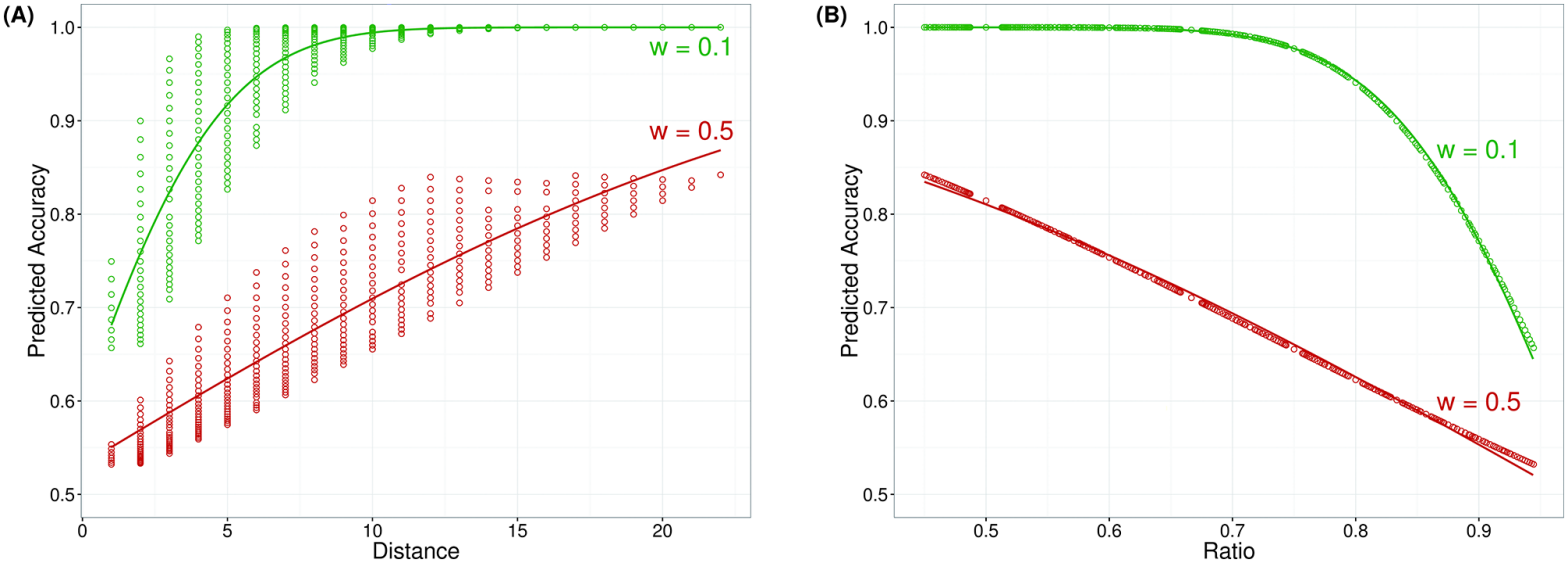
Interrelations of accuracy based measures. Dots reflect predicted accuracy for a Weber fraction of 0.1 (i.e., more accurate ANS, depicted in green) and 0.5 (less accurate ANS, given in red) calculated according to the linear model and based on the item set used in our experiments. In panel (A) accuracy differed for a given numerical distance, because in our item set several ratios for a given distance were included. The lines in panel (A) reflect the numerical distance effect calculated using a generalized linear model with distance as predictor and the predicted accuracy as dependent variable, resulting from a Weber fraction of 0.1 (green) or 0.5 (red). The lines in panel (B) reflect the ratio effect, again calculated using a generalized linear model with ratio as predictor and predicted accuracy as dependent variable.

Moreover, it can be concluded–based on the mathematical considerations above–that mean accuracy must be positively related to NDE_acc and negatively to NRE_acc. The interrelations of the accuracy based measures are also illustrated in Fig 2 showing the NDE_acc and the NRE_acc for both a hypothetical participant with a relatively small Weber fraction (i.e., a more accurate ANS; depicted in green) and a participant with a larger Weber fraction (i.e., less accurate ANS; depicted in red).

Taken together, all accuracy based measures should be strongly related–both from a theoretical and from a mathematical point of view. However, recent studies investigating the relationship between accuracy based ANS measures questioned this assumption [31,39,40]. Although these studies reported high correlations between mean accuracy and the Weber fraction, the correlations between mean accuracy as well as the Weber fraction and the NRE_acc were only small to moderate [59] (the terms numerical distance effect and numerical ratio effect were sometimes used interchangeably, e.g., [39]). These results from the literature contradict the common hypothesis that all accuracy based measures reflect the same concept (i.e., ANS acuity). Based on these results, it was postulated recently that the NRE_acc–despite its theoretical link to the ANS–may not necessarily reflect ANS acuity [40,46]. This conclusion leads to the following questions: (1) how to interpret the results of previous studies using NRE_acc, when assuming that this measure may not reflect ANS acuity and (2) what is the actual conceptual meaning of NRE_acc.

Low correlations between NRE_acc and other ANS measures, however, might also be explained by psychometric properties of the measure, especially its (low) reliability [39,40]. Poor reliability limits the potential size of observed correlations [60]. Thus, the correlation between NRE_acc and other accuracy based measures might have been low, because of lower reliability of NRE_acc as compared to the reliability of the Weber fraction or mean accuracy [39,40]. Until this methodological alternative explanation for low correlations between NRE_acc and other accuracy based measures is evaluated, it is still possible that all accuracy based measures are indeed related and commonly reflect ANS acuity. In our first experiment, we aimed at investigating this issue. Therefore, we examined the relationship between all accuracy based measures, trying to avoid the potential confound of poor reliability of the measures, especially of NDE_acc and NRE_acc. To do so, we employed a large number of trials (n = 400), following the recommendations of Lindskog and colleagues [39].

### RT based measures

Besides accuracy based measures also RT based measures were often used to assess ANS acuity [30,36,41,42,61–63]. RT based measures include mean RT as well as the numerical distance and ratio effect based on RT data (NDE_RT/ NRE_RT). They are often employed in addition to accuracy based measures [52,62,63] but also as the unique measure [41,42,44]. Mean RT is used as an index of ANS acuity, as it is assumed that participants with more accurate ANS representations respond faster than participants with a less accurate ANS [45,62,64]. The NDE_RT (resp. NRE_RT) describes the finding that RT increases as the numerical distance (ratio; i.e., smaller numerosity divided by larger numerosity) between two to-be-compared numbers decreases (resp. increases) [27]. The NDE_RT and the NRE_RT are calculated using linear regression analyses with RT as dependent variable and numerical distance or numerical ratio between the two numerosities as predictor variable. The resulting slope for distance/ ratio reveals the NDE_RT/ NRE_RT. Both a smaller NDE_RT and a smaller NRE_RT have been assumed to reflect a more accurate ANS [30,45,65].

NDE_RT and NRE_RT are inversely related, because distance and ratio are inversely related (in our experiments: *r* = -.898 and *r*_*s*_ = -.911) and both measures are calculated analogously. The relationship between mean RT and these measures cannot be derived analytically. However, there are hints from the literature that NDE_RT (distance defined as D = |n_1_ –n_2_|) and mean RT are negatively related [30]. Holloway and Ansari (2009) reported a positive relationship between NDE_RT and mean RT. However, in their study NDE_RT was defined as smaller distance minus larger distance resulting in negative values. Thus, the coding is reversed compared to the coding in the present experiments. Therefore, we expected a negative correlation between NDE_RT and mean RT. Consequently, because NDE_RT and NRE_RT are inversely related, there should be a positive relationship between NRE_RT and mean RT. However, the relationship between these RT based measures has not been investigated systematically so far. Thus, in the present experiment, we will also focus on the relationship between RT based measures.

### Interrelations between accuracy and RT based measures

There are two opposing positions regarding the relationship between accuracy and RT based measures: On the one hand, it has been proposed that a more accurate ANS is reflected in both more accurate discrimination performance (i.e., higher mean accuracy) and faster RT (i.e., smaller mean RT; [45,52,62,66]). Thus, these measures should be negatively related. On the other hand, participants can show a speed-accuracy trade-off. Thus, they may prefer accuracy over speed resulting in more accurate performance (i.e., higher mean accuracy) and slower RT (i.e., higher mean RT) or vice versa [67,68]. In case of a speed-accuracy trade-off, accuracy and RT are positively related [66].

Assuming that both accuracy and RT indicate ANS acuity in a similar way (i.e., correspond in assessing the ANS and, therefore, are negatively related), both accuracy and RT based measures have been employed to assess ANS acuity in numerous studies [30,31,39,40,45,46,48]. However, accuracy and RT do not necessarily reflect the same underlying processes [69]. Thus, it is important to investigate empirically, whether these measures can indeed be used interchangeably. Previous studies investigated the relationship between NRE_RT and the accuracy based measures mean accuracy, Weber fraction as well as NRE_acc and reported only low or moderate correlations [31,39,40]. Their results challenged the assumption that accuracy and RT based measures both reflect ANS acuity in a comparable way. However, these studies reported poor reliabilities for NRE_RT. Thus, low correlations with accuracy based measures might (again) be explained by poor reliability of the NRE_RT in the respective studies. In our first experiment, we investigated this issue by examining the relationship between accuracy and RT based measures with presumably sufficient reliability due to a higher number of items.

Importantly, if participants show a speed-accuracy trade-off, this will result in an ambiguous, inconsistent interpretation of the measures accuracy and RT: higher mean accuracy is commonly interpreted as an index for higher ANS acuity, whereas larger mean RT is commonly interpreted as an index for lower ANS acuity. Thus, both measures provide opposing information regarding the acuity of the underlying ANS representations. In this case, it remains unclear, which measure allows conclusions regarding ANS acuity–accuracy or RT based measures.

Sequential sampling models provide an account to describe how decisions between two alternative responses are reached and to explain a speed-accuracy trade-off [70,71]. These models–in particular random walk or diffusion models–have already been applied in the context of numerical comparison or discrimination [27,66,72] and have also been employed for non-symbolic dot comparison [28,73]. According to these models the representation of stimuli in the human brain is noisy. To make a decision, information needs to be accumulated over time until a criterion is reached (i.e., sufficient evidence for a decision has accumulated) and the response can be executed [71,74]. According to a diffusion model [74,75], task performance depends on two factors: First, task performance depends both on properties of the stimuli varying between trials (e.g., stimulus difficulty), and on the neural processing mechanisms varying between participants (e.g., ANS acuity). The rate of accumulation of information (i.e., the drift rate *v* in Ratcliff’s diffusion model) was proposed to reflect differences in trial difficulty [74,76] and, therefore, should vary depending on the representational overlap between the to-be-compared numerosities. For example, the comparison of 5 versus 10 dots (i.e., small overlap) should trigger a faster decision than the comparison of 9 versus 10 dots (i.e., larger overlap). In turn, this should result in a higher drift rate for the comparison of numerosities with small representational overlap. In contrast, for more difficult comparisons (i.e., larger overlap), more iterations are required until a decision can be made (i.e., the drift rate is smaller). Accordingly, RTs are longer and accuracy is reduced for more difficult comparisons [71,74].

Second, performance is also influenced by the amount of information needed to make a decision reflecting the participants’ individual decision criterion [71]. Differences in decision criteria (i.e., the amount of evidence needed for a decision) can explain speed-accuracy trade-offs and are accounted for in the diffusion model by varying the response threshold *a*. Reducing this response threshold leads to faster RT but also reduces accuracy, reflecting the performance of a participant favoring speed over accuracy. In contrast, a high response threshold results in fewer errors, but longer response times. These preferences can reduce or even conceal differences in difficulty (i.e., the overlap of the tuning curves; see [66]): If a participant prefers speed over accuracy (resulting in overall faster response times and higher error rates), the influence of difficulty (i.e., overlap of tuning curves) on RT will be smaller than if a participant prefers accuracy over speed. In contrast, for a participant who favors speed over accuracy, error rates will vary more reliably with difficulty (i.e., overlap of tuning curves) than for participants favoring accuracy. Thus, the diffusion model provides hypotheses under which condition (i.e., the preferences for accuracy or speed) accuracy or RT should be more informative about the underlying ANS representation. We will investigate these hypotheses in two experiments, where we systematically manipulated participants’ preferences for accuracy or speed.

### Composite measures

Measures combining RT and accuracy data have also been proposed in the literature. For example, the inverse efficiency score was introduced as a measure of ANS acuity [36,43,77] and was proposed to control for potential speed-accuracy trade-off ([77,78] but see [79]). This score can be calculated by dividing mean RT of correct responses by the proportion of correct responses [79,80]. The efficiency score is given in milliseconds and can be interpreted like mean RT (i.e., the smaller the efficiency score, the higher ANS acuity [77]). Nevertheless, studies investigating the relationship between the efficiency score and the other ANS measures are missing so far.

## Overview

In the present study, we systematically investigated the relationship between commonly used ANS measures including accuracy based measures, RT based measures, and composite measures, in order to clarify, whether these measures may be used interchangeably. We tried to avoid the potential confound of poor reliability, which reduces the potential size of correlations and might have caused low correlations between some of the measures in previous studies. Therefore, we employed a large number of trials to increase reliability.

Moreover, we specifically focused on the direction of the relationship between accuracy and RT based measures. Accuracy and RT based measures have often been postulated to correspond in assessing ANS acuity [45,52,62,66]. However, participants might also trade speed against accuracy. The latter would be problematic as in this situation accuracy and RT based measures may result in opposite conclusions regarding ANS acuity, because higher mean accuracy–indicating a more accurate ANS–is associated with larger RTs–indicating a less accurate ANS. This raises the question, which measure provides information about the underlying ANS representations.

Therefore, in our second and third experiment we investigated, whether accuracy or RT is more informative about the underlying ANS representations depending on participants’ preferences for accuracy or speed. To do so, we manipulated individual preferences for accuracy or speed and examined the part of variance in accuracy/ RT data explained by the ratio between the to-be-compared numerosities as an indicator for the underlying ANS representations. In Experiment 2, we explicitly instructed the participants to respond either (1) as quickly as possible (speed instruction), (2) as accurately as possible (accuracy instruction), (3) or as quickly and as accurately as possible (instruction emphasizing on both, speed and accuracy). In our third experiment, we manipulated the preference for speed or accuracy implicitly by varying presentation duration of the stimuli from very short (i.e., 50ms or 200ms) to longer (i.e., 2400ms) and even unlimited (i.e., self-paced) presentation durations.

## Experiment 1

In Experiment 1, we evaluated the relationship between (1) all accuracy based measures and (2) between all RT based measures as well as (3) the relationship between accuracy based, RT based, and composite measures. If all these measures indeed reflect ANS acuity in a comparable way, they should be correlated substantially. Our assumptions regarding size and direction of the correlations–based on the mathematical considerations in the introduction and previous empirical findings–are summarized in Table 1. We were particularly interested in whether the relationship between accuracy and RT based measures was negative (indicating that a more precise ANS is reflected in more accurate performance and faster RTs) or positive (indicating a speed-accuracy trade-off). As previous studies used accuracy and RT based measures interchangeably, assuming that these measures correspond in assessing ANS acuity, we formulated the hypotheses depicted in Table 1 in line with this assumption. We reported Spearman correlations, a measure of a monotonous relationship, as we expected non-linear relationships between most of the measures. In case of a speed-accuracy trade-off, the direction of the correlations across accuracy and RT measures should be reversed. To overcome the potential confound of poor reliability, which reduces the correlation between the measures, we employed a larger number of trials following the recommendation of Lindskog and colleagues [24].

**Table 1:** Expected Spearman correlations between all ANS measures based on mathematical considerations (in bold) and the assumption that accuracy and RT correspond in assessing ANS acuity.

ANS measure	Mean acc	NDE_acc	NRE_acc	Weber fraction	Mean RT	NDE_RT	NRE_RT	Efficiency
Mean acc	1							
NDE_acc	Positive	1						
NRE_acc	Negative	**-.911**	1					
w	**-1**	**-.911**	**1**	1				
Mean RT	Negative	negative	positive	positive	1			
NDE_RT	positive	positive	negative	negative	negative	1		
NRE_RT	Negative	negative	positive	positive	positive	**-.911**	1	
Efficiency	positive	unclear	unclear	unclear	positive	unclear	unclear	1

*Note*. Mean acc = mean accuracy, NDE_acc = numerical distance effect calculated based on accuracy data, NRE_acc = numerical ratio effect calculated based on accuracy data, w = Weber fraction, Mean RT = mean response time, NDE_RT = numerical distance effect calculated based on RT data, NRE_RT = numerical ratio effect calculated based on RT data, Efficiency = inverse efficiency score.

### Materials and Methods

#### Participants

Sixty-one adult participants (37 female, 3 left-handed, *M*_*age*_ = 24.66 years, *SD*_*age*_ = 3.30, *age range* = 18-33 years) were included in the experiment. All participants provided written informed consent prior to their participation and received a compensation of 8€ per hour. The experiment was approved by the local ethics committee of the Leibniz-Institut fuer Wissensmedien in Tuebingen.

#### Stimuli and procedure

We administered a non-symbolic dot comparison task to assess ANS acuity. Participants were instructed to indicate by key press, which of two sets of dots presented simultaneously contained more dots. When the left dot set was more numerous they should press the left response key (left Ctrl key on a standard QWERTZ keyboard), whereas they should press the right response key (right Ctrl) when the right dot set was more numerous. Participants were instructed to “respond as quickly and accurately as possible”. The two sets were presented as black dots against a white background and were separated by a black line in the middle of the screen. At a viewing distance of approximately 60cm dot sets extended to a visual angle of 2.7°- 19.4°horizontally and 4.7°-15.4°vertically. Stimuli were displayed for 200ms, followed by a white screen, which remained visible until participants responded. Prior to the presentation of the stimuli a fixation mark (i.e., a black square) was displayed in the middle of the screen for 500ms. The number of dots in each set varied between 10 and 40 with the following ratios: 0.5, 0.6, 0.7, 0.8 and 0.9. There were 80 trials per ratio totaling in 400 experimental trials, which were presented in random order. Whether the larger dot set was presented on the left or right side was counterbalanced for each participant. Before the experimental trials participants completed 5 practice trials. The dot sets were created using the Matlab script of Gebuis and Reynvoet [81] to control for visual properties. In all experiments of the current study the same dot sets were used for all participants.

#### Analysis

First, we calculated the following indices of ANS acuity separately for each participant: mean accuracy, Weber fraction (both from the linear model, the logarithmic model, and the modified logarithmic model), NDE_acc, NRE_acc, mean RT, NDE_RT, NRE_RT, and the inverse efficiency score. RT was defined as the time between the end of stimulus presentation and the participant’s response (i.e., key press). Before calculating the measures based on RT data, a trimming procedure excluded all RTs deviating more than 3 *SD* from the individual participant’s mean. This led to a loss of less than 1.8% of the data. Mean accuracy and mean RT were calculated aggregating over all ratios. The results on mean RT reported in the present experiments are based on all responses (i.e., correct and incorrect responses). However, mean RT_all_ (based on all responses) and mean RT_correct_ (based on correct responses only) were highly correlated at *r* = .998 and therefore can be used interchangeably. Consequently, the same pattern of results was also found when incorporating only RTs of correct responses.

NDE_acc and NRE_acc were calculated per participant using a generalized linear model with a binomial error distribution and the probit as link function. The probit function models the sigmoidal pattern of proportional data. We used a binomial error distribution for the generalized linear model to account for the fact that the error variance of accuracy data may be reduced near the upper (i.e., 1) or the lower bound (i.e., 0). Accuracy served as the dependent variable while the numerical distance between the to-be-compared numerosities and the numerical ratio between the smaller and the larger numerosities, respectively, were the predictor variables. NDE_RT and NRE_RT were calculated using linear regression analyses with RT as dependent variable and the numerical distance or the numerical ratio between the two numerosities as predictor variables.

The individual Weber fractions for the linear model were estimated relying on Eq 5.

Thus, we used a probit model with the parameter β = 1/w (and hence, w = 1/ β), as the probit model takes the form of: Pr(Y = 1|X_i_ = x_i_) = Φ(x_i_β) with Pr indicating the probability of a correct answer (i.e., 1 for correct and 0 for incorrect answer) [82]. In the above formula (Eq 5) ${\text{x}}_{\text{i}}\;=\;\frac{\left|{\text{r}}_{\text{i}}\;-\;1\right|}{\sqrt{{\text{r}}_{\text{i}}{}^{2}\;+\;1}}$. Accordingly, we fitted Weber fractions per individual participant, using a generalized linear model with a binomial error distribution and the probit as link function. Accuracy was the dependent variable and $\frac{\left|\text{r }-\;1\right|}{\sqrt{{\text{r}}^{2}\;+\;1}}$ was the predictor in the model. The resulting *β*s were transformed into Weber fractions w by calculating w = 1/β. This model does not include an intercept in contrast to the models for the NDE_acc and the NRE_acc.

To calculate the Weber fractions for the logarithmic model we used a similar approach. In this case we employed Eq 6. Thus, we used a probit model with the parameter $\beta =1/(w\sqrt{2})$ and the predictor log_2_(*r*_*num*_). Therefore, we fitted Weber fractions per participant, using a generalized linear model with a binomial error distribution, the probit as link function, accuracy as dependent variable and log_2_(*r*_*num*_) as predictor. The resulting *β*s were transformed into Weber fractions w by calculating $w=\;1/(\sqrt{2}\beta )$.

Finally, we also fitted Weber fractions for the modified logarithmic model [55], relying on Eq 7. Thus, we used a probit model with the parameter $\beta =1/(w\sqrt{2})$ and the predictors log_2_(*r*_*num*_), log_2_(*r*_*size*_), and log_2_(*r*_*spacing*_). Therefore, we fitted Weber fractions per participant, using a generalized linear model with a binomial error distribution, the probit as link function, accuracy as dependent variable and, , and log_2_(*r*_*num*_), log_2_(*r*_*size*_), and log_2_(*r*_*spacing*_) as predictors. This model again does include an intercept. The resulting *β*s were transformed into Weber fractions w by calculating $w=\;1/(\sqrt{2}\beta )$. Importantly, we excluded all participants for which the predictor β was not significantly different from zero, irrespective of which model was used to estimate the Weber fraction (alpha level = .05; this affected 7 participants). Note that this approach is similar to excluding participants with a negative Weber fraction (which is theoretically not possible) and a very large Weber fraction (i.e., w > 2).

The efficiency score was calculated by dividing mean RT of correct responses by the proportion of correct responses [79]. However, this efficiency score weights accuracy in a non-linear fashion. Whether or not this assumption is plausible to make, was not yet verified. Therefore, as suggested by an anonymous reviewer, we additionally calculated another composite score by *z*-transforming mean accuracy and mean RT separately before averaging these two values (subsequently referred to as *z*-score). This procedure ensures an equal variance contribution from both accuracy and RT to the composite score.

In a second step, we calculated the split-half reliability for each measure, in order to ensure that the measures were sufficiently reliable. This is an important prerequisite, as it allows to rule out the possibility that low correlations between some of the measures are simply due to poor reliabilities [60]. To calculate the split-half reliabilities, we correlated the ANS measures computed based on one half of the trails with the ANS measures computed based on the other half. The two halves were randomly selected. To get a good estimate of the reliability, we repeated this procedure 100 times and averaged the resulting 100 split-half reliabilities. Split-half reliabilities were Spearman-Brown corrected for the reduced test length by using the R package CTT [83–85].

Next, a correlation analysis was conducted to investigate the relationship between the ANS measures. The Shapiro-Wilk test revealed that six out of the eleven ANS measures (i.e., NDE_acc, NDE_RT, NRE_RT as well as the Weber fraction calculated both from the linear, the logarithmic, and the modified logarithmic model) deviated significantly from a normal distribution [86]. Therefore, Spearman's rank correlation coefficients were calculated using the R package Hmisc [87]. To correct for multiple comparisons, when testing for departure from zero, the Benjamini-Hochberg procedure was applied [88]. Please note, the raw data for all three experiments can be found in the Supporting Information (see S1 File for demographic data and S2 File for experimental data).

### Results

#### Reliability

Descriptive statistics, including mean (M), standard deviation (SD), minimum (Min) and maximum (Max), as well as split-half reliabilities of all ANS indices are shown in Table 2. Importantly, all accuracy based measures as well as mean RT and the composite scores were highly reliable. Our results also revealed moderate to high split-half reliabilities of NDE_RT and NRE_RT according to the classification of Murphy and Davidshofer [89]. These results enable us to investigate the relationship between the ANS measures (especially between NDE/ NRE and the other measures) ruling out the possible alternative explanation that low correlations might be simply caused by poor reliabilities.

**Table 2:** Descriptive statistics and split-half reliabilities of all ANS measures.

ANS measure	*M*	*SD*	Min	Max	Split-half (uncorrected)	Split-half (Spearman-Brown corrected)
Mean acc in %	67.08	9.02	49.25	82.75	0.95	0.97
NDE_acc	0.04	0.03	-0.02	0.13	0.85	0.92
NRE_acc	-1.58	1.00	-3.99	0.63	0.83	0.91
w (lin)	0.61	0.35	0.24	1.79	0.87	0.93
w (log)	0.96	0.55	0.36	2.91	0.87	0.93
w (mod.log)	0.52	0.31	0.25	1.78	0.90	0.95
Mean RT in ms	481.87	199.22	104.52	1078.52	1.00	1.00
NDE_RT in ms	-2.46	4.23	-16.56	10.16	0.77	0.87
NRE_RT in ms	46.48	135.75	-320.90	533.24	0.74	0.85
Efficiency in ms	701.27	250.58	235.08	1391.73	0.99	1.00
*z*-score	0.00	0.88	-1.94	1.94	0.98	0.99

*Note*. Mean acc = mean accuracy, NDE_acc = numerical distance effect calculated based on accuracy data, NRE_acc = numerical ratio effect calculated based on accuracy data, w = Weber fraction calculated from the linear model (lin), the logarithmic model (log) or the modified logarithmic model (mod.log), Mean RT = mean response time, NDE_RT = numerical distance effect calculated based on RT data, NRE_RT = numerical ratio effect calculated based on RT data, Efficiency = inverse efficiency score, % = percentage correct.

### Correlation analysis

In a next step, we investigated the relationship between the ANS measures by calculating all pairwise correlations. The results of the correlation analysis are reported in Table 3 showing the Spearman correlation coefficients between all ANS measures.

**Table 3:** Spearman correlation coefficients between all ANS measures.

	(1)	(2)	(3)	(4)	(5)	(6)	(7)	(8)	(9)
(1) Mean acc	1.00								
(2) NDE_acc	.88***	1.00							
(3) NRE_acc	-.90***	-.97***	1.00						
(4) w	-.99***	-.89***	.91***	1.00					
(5) w (mod.log)	-.76***	-.68***	.73***	.78***	1.00				
(6) Mean RT	.51***	.41***	-.38***	-.37***	-.40***	1.00			
(7) NDE_RT	-.56***	-.46***	.46***	.54***	.48***	-.48***	1.00		
(8) NRE_RT	.37***	.34*	-.35***	-.35*	-.28*	.23	-.81***	1.00	
(9) Efficiency	.22	.14	-.12	-.08	-.18	.93***	-.34***	.13	1.00
(10) z-score	.87***	.73***	-.73***	-.81***	-.68***	.84***	-.59***	.31*	.62***

Note.

* *p* < .05,

*** *p* < .001;

*p*-values were adjusted for multiple testing using the Benjamini-Hochberg procedure. Cells were colored grey whenever the direction of the correlations was opposite to our predictions from Table 1. Mean acc = mean accuracy, NDE_acc = numerical distance effect calculated based on accuracy data, NRE_acc = numerical ratio effect calculated based on accuracy data, w = the Weber fraction estimated using the linear or the logarithmic model, w (mod.log) = the Weber fraction estimated using the modified logarithmic model, Mean RT = mean response time, NDE_RT = numerical distance effect calculated based on RT data, NRE_RT = numerical ratio effect calculated based on RT data, Efficiency = inverse efficiency score. Note that the Weber fractions estimated using the linear model correlated at *r*_*s*_ = 1 with the Weber fractions estimated using the logarithmic model and, therefore, are displayed in one row/column.

**Fig 3:**
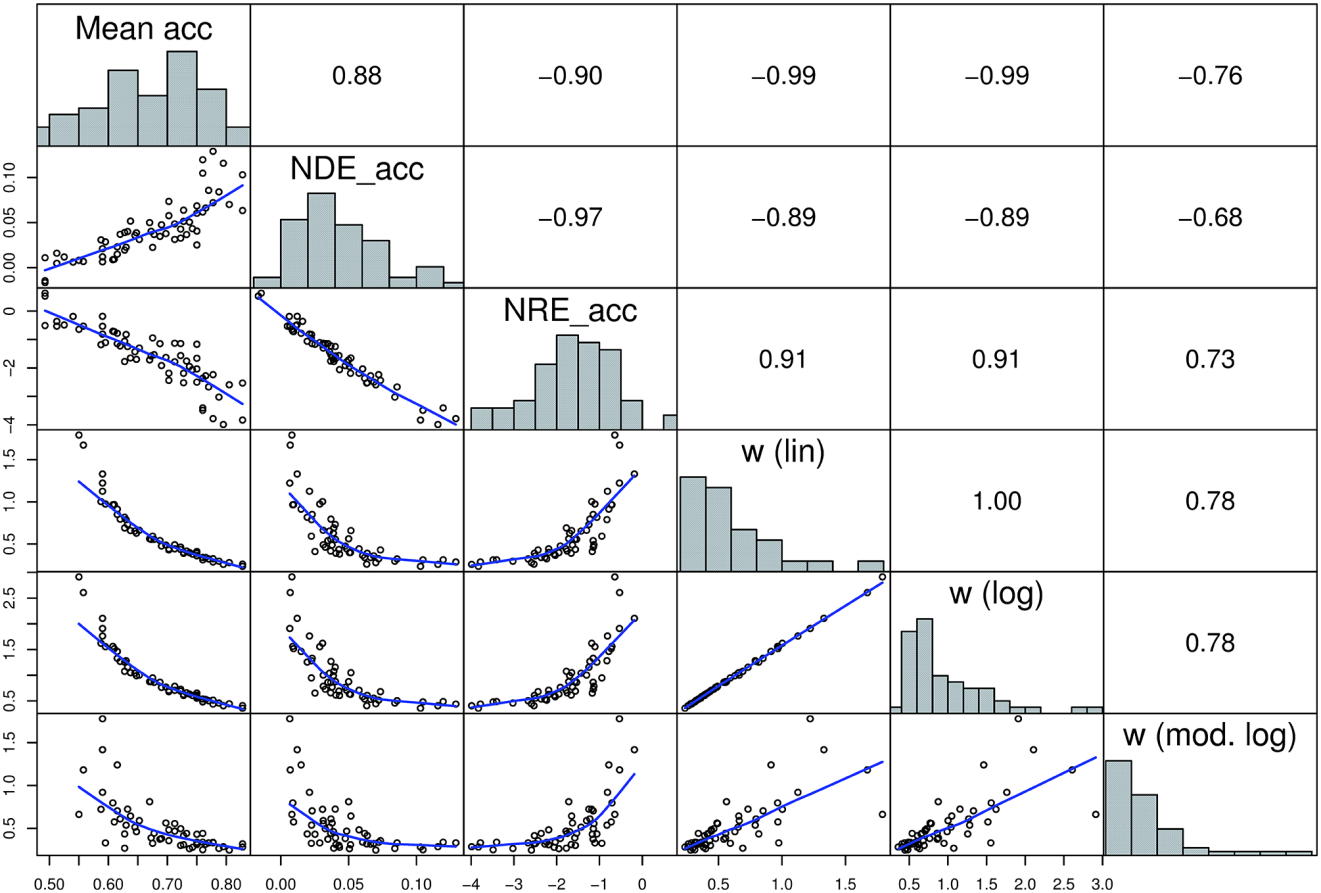
Distributions and interrelations of accuracy based measures. Histograms on the diagonal show the distribution of the measures. Scatterplots in the lower panel represent the relationship between the respective measures. Blue line shows the “lowess” curve (calculated using the LOWESS smoother which uses locally-weighted polynomial regression). In the upper panel the respective Spearman correlation coefficients are shown.

Given high reliability for all accuracy based measures, all these measures were highly correlated. Moreover, the direction of the correlations between the accuracy based measures was in line with the assumptions based on the mathematical relation between ratio, distance, and the Weber fraction (see also Fig 3): the NDE_acc and NRE_acc were negatively related. Moreover, mean accuracy correlated positively with NDE_acc and negatively with the other accuracy based measures. In addition, the Weber fraction was positively related to NRE_acc and negatively related to NDE_acc. Importantly, the Weber fraction calculated from the modified logarithmic model, which accounts for visual properties of the stimuli and, therefore, was proposed to be a theoretically more valid measure of ANS acuity [55], was highly correlated with all other accuracy based measures. However, the correlations were slightly smaller, which may be explained by the fact that the Weber fractions calculated from the modified logarithmic model are corrected for influences of visual properties.

**Fig 4:**
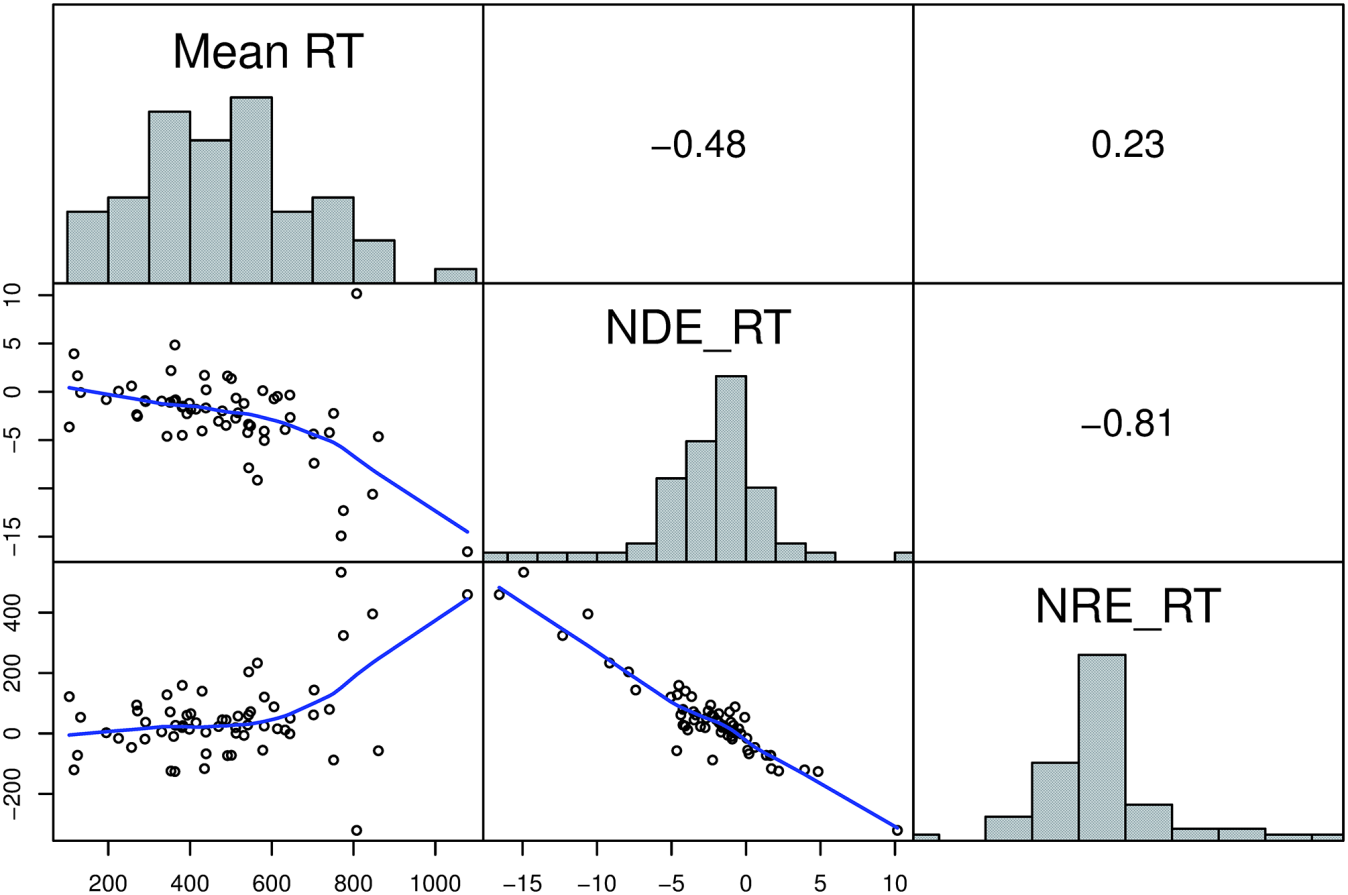
Distributions and interrelations of RT based measures. Histograms on the diagonal show the distribution of the measures. Scatterplots in the lower panel represent the relationship between the measures. Blue line shows the “lowess” curve (calculated using the LOWESS smoother which uses locally-weighted polynomial regression). In the upper panel the respective Spearman correlation coefficients are shown.

Despite moderate to high reliability, our results revealed that the correlations between RT based measures were substantially lower than the correlations between accuracy based measures (see Table 3). Moreover, RT based measures were not as homogenous as accuracy based measures. As expected, NDE_RT and NRE_RT were highly correlated. In contrast, the correlations between mean RT and these two measures were substantially lower (see also Fig 4). Finally, the direction and the size of the correlation between mean RT and NDE_RT was in line with our expectations based on previous findings [30].

**Fig 5:**
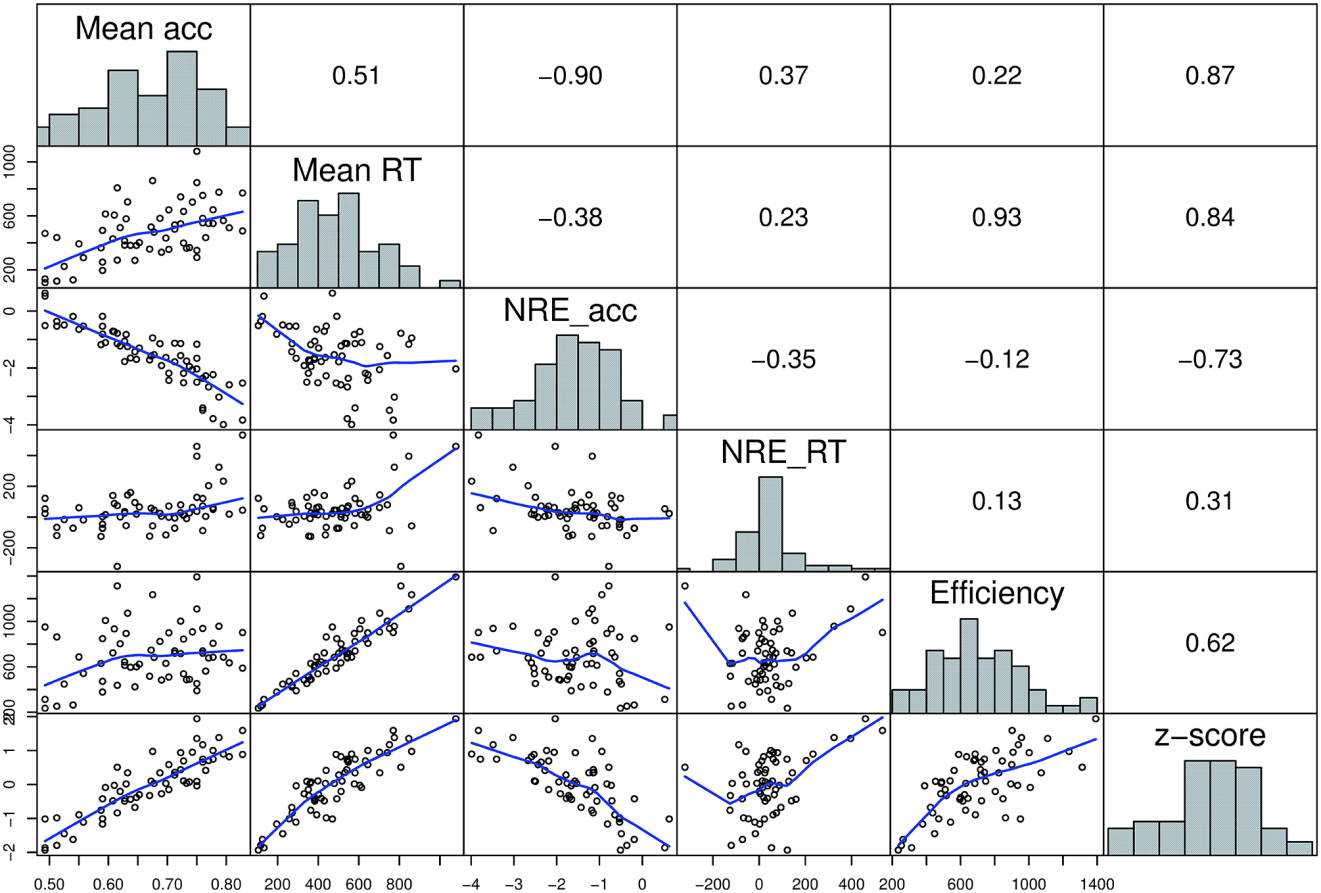
Interrelations across selected accuracy based, RT based measures and composite scores. Histograms on the diagonal show the distribution of the measures. Scatterplots in the lower panel represent the relationship between the measures. Blue line shows the “lowess” curve (calculated using the LOWESS smoother which uses locally-weighted polynomial regression). In the upper panel the respective Spearman correlation coefficients are shown.

We were particularly interested in the direction of the relationship between accuracy and RT based measures. We found a significant positive correlation between mean accuracy and mean RT (see Fig 5). Therefore, the observed direction of the correlations across accuracy and RT based measures was the reversed from what we expected in Table 1 (based on the assumption that accuracy and RT correspond in assessing ANS acuity, i.e. are negatively related). Thus, the distance and ratio effects based on accuracy and RT were negatively correlated (Table 3). Moreover, the size of the correlations across accuracy and RT based measures were mainly of medium size ([59] see Table 3 and Fig 5). Furthermore, the direction of the correlations between mean accuracy and NDE_acc is in the opposite direction as the correlation between mean RT and NDE_RT (analogue pattern for the ratio effect).

Regarding the relationship between composite scores and the other ANS measures, it is striking that the efficiency score was highly correlated with mean RT, but did not correlate with any accuracy based measure. In contrast to the efficiency score, the *z*-score was indeed highly correlated both with accuracy based measures and mean RT and, therefore, might constitute a better composite measure (Fig 5).

### Discussion

So far, different measures were employed to index ANS acuity. Some of these measures were calculated based on accuracy data, whereas other measures were based on RT data or also combined both types of information (i.e., the efficiency score). The use of these different measures is based on the assumption that all these measures reflect ANS acuity in a similar way. Previous studies provided first evidence contradicting this assumption by revealing only small to moderate correlations among accuracy based measures as well as between accuracy and RT based measures. More precisely, weak correlations were found between the NRE (based on accuracy or RT data) and mean accuracy as well as the Weber fraction [31,39,40]. However, these low correlations might also be explained by the low reliability of the NRE, which restricts the obtainable size of correlations [60]. In the present experiment, we examined the interrelation between ANS measures while ensuring sufficient reliability. To do so, we employed a large number of trials (i.e., 400 trials) as recommended by Lindskog and colleagues [39], who conducted a simulation study to estimate the number of trials necessary for sufficient reliability. In line with this, our results revealed high split-half reliabilities coefficients for all accuracy based measures and moderate to high reliabilities for all RT based measures. This was an important prerequisite for the evaluation of the interrelation of ANS measures ruling out the potential alternative explanation that low correlations might be driven by poor reliability.

Our results revealed that–when employing sufficiently reliable measures–all accuracy based measures (i.e., mean accuracy, Weber fraction, NDE_acc, and NRE_acc) were indeed highly correlated. This is in line with the common assumption that all accuracy based measures assess the same underlying concept (i.e., ANS acuity) and, thus, can be used interchangeably. Our results support the view that poor reliabilities reduced the expected correlations between accuracy based measures in previous studies. In the present experiment, the measures were substantially more reliable than in previous studies [39,40]. Hence, poorer reliability of NRE_acc in previous studies might indeed have reduced the correlation between NRE_acc and the other accuracy based measures. This finding further implies that when researchers assume that accuracy based measures are equivalent, they need to ensure sufficient reliability and, thus, it is necessary to employ a very large number of trials. However, so far it is rather common to use less than 100 trials [2,30,36,51,53,61,64,90–94], which is substantially below the recommended 400 trials proposed by Lindskog and colleagues (2013) [39].

Moreover, our results revealed that RT based measures were not as homogenous as accuracy based measures and cannot be considered to form one coherent category. While all accuracy based measures were highly correlated amongst each other, the correlations between RT based measures were substantially lower: only NDE_RT and NRE_RT were strongly related, but correlated only moderately with mean RT. Thus, our results revealed that not all RT based measures seem to assess the same underlying concept to the same extent.

We also focused on the interrelations between accuracy and RT based measures. Despite better reliabilities of the measures in the present experiment the correlations between NRE_RT and the accuracy based measure Weber fraction as well as mean accuracy were essentially comparable to the results of previous studies [31,39,40]. This indicates that these correlations were not artificially reduced by poorer reliabilities. Only the correlation between NRE_acc and NRE_RT was substantially larger in the present experiment than in previous studies, which might be attributed to better reliabilities.

**Fig 6:**
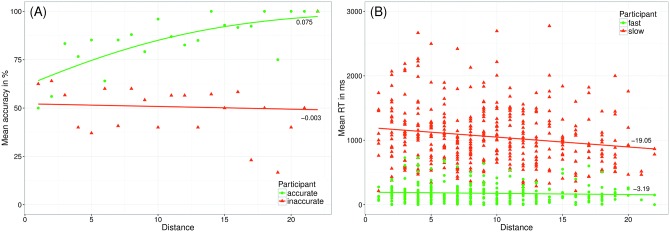
Relationship between distance effects and mean accuracy/ mean RT. Performance [accuracy (panel A) and speed (panel B)] of an exemplary participant performing good (i.e., either accurate or fast; depicted in green) and an exemplary participant performing poor (i.e., either inaccurate or slow; depicted in red). Lines reflect the distance effects calculated using a generalized linear model for accuracy data and a linear model for RT data.

The direction of the correlations between mean accuracy and NDE_acc is opposed to that of correlation between mean RT and NDE_RT (similar pattern for the ratio effect). This pattern of results can be explained by the reversed coding of task performance in accuracy and RT, because better task performance is reflected in larger mean accuracy, but smaller RT. This also affects the direction of the distance effect: for accuracy data the slope is positive, whereas for RT data the slope is negative (see Fig 6). Moreover, for accuracy task performance is restricted downwards due to chance level, which leads to a flat slope for participants performing very poorly (i.e., a floor effect). In contrast, participants performing more accurately show a larger distance effect–resulting in a positive correlation between mean accuracy and NDE_acc. In contrast, RTs are restricted upwards, because participants cannot respond unlimitedly fast. Hence, the NDE_RT resulting for participants performing very fast is flat. Participants performing more slowly show a steeper (negative) slope. As the slope is negative, the parameter reflecting NDE_RT is smaller, which results in a negative correlation with mean RT (i.e., smaller value for larger NDE_RT and larger values for larger mean RT). Most importantly, this pattern contradicts the common expectation that a more accurate ANS corresponds to smaller effects of distance (or ratio) irrespective of being calculated from accuracy or RT data [30,45]. In contrast, a smaller distance effect (i.e., flat slope) does not necessarily indicate more precise ANS representations, because a flatter slope can either result from a particular poor performance (in case of accuracy data) or very good performance (for RT data). The same rationale also holds for the ratio effect. However, note that distance and ratio effects are inversely related.

More importantly, we found a positive correlation between mean RT and mean accuracy, providing evidence for a speed-accuracy trade-off. These results contradict the assumption that accuracy and RT based measures correspond in assessing ANS acuity, because in this case mean accuracy and mean RT should be negatively related. A speed-accuracy trade-off leads to diverging conclusions about ANS acuity and ambiguous interpretations, as more accurate performance has been interpreted as an indicator for higher ANS acuity, whereas longer RTs have been assumed to reflect less accurate ANS acuity (and vice versa). Thus, it remains unclear, which measure allows for valid conclusions about the underlying representations.

To control for speed-accuracy trade-offs some studies used efficiency scores, which combine accuracy and speed in one measure [43,77]. However, the efficiency score was not correlated with any of the accuracy based measures, and, instead correlated highly with mean RT only. This clearly contradicts previous assumptions that the inverse efficiency score reflects both accuracy and speed of task performance. Moreover, this pattern of results questions the use of the inverse efficiency score as a composite measure being suited to control for a speed-accuracy trade-off by considering both accuracy and speed. However, the *z*-score appeared to constitute such a measure, which might control for a speed-accuracy trade-off, as this score was highly correlated with both accuracy and RT measures.

To sum up, based on the present findings, accuracy and RT based measures do not seem to correspond in measuring ANS acuity. This is an important finding, because numerous studies did use these measures interchangeably as an index of ANS acuity [30,36,41,42,61–63]. Instead, our data support the notion that participants show a speed-accuracy trade-off, which raises the question which measure is informative about the underlying representation. The *z*-score combining both accuracy and RT might be helpful in this respect. Another possible approach addressing the issue of speed-accuracy trade-off refers to the diffusion model, which proposed that—depending on the individual preferences for accuracy or speed–either accuracy or RT measures may be more informative about the underlying representation. We evaluated this issue in two further experiments.

## Experiment 2

In our second experiment, we aimed at investigating accuracy and RT measures in the case of a speed-accuracy trade-off. In particular, we were interested under which conditions accuracy or RT based measures were more informative about the underlying ANS representations.

Pursuing this aim, we employed a non-symbolic dot comparison task with three different instructions which varied regarding the degree to which they emphasized speed or accuracy. Participants were instructed to respond either (1) as quickly as possible (speed instruction), (2) as accurately as possible (accuracy instruction), (3) or as quickly and as accurately as possible (combined instruction). This method was used frequently to induce a speed-accuracy trade-off [67] and allowed us to explicitly manipulate individual preferences for speed or accuracy. The diffusion model provides not only an account to explain a speed-accuracy trade-off, but also offers a theoretical framework to derive assumptions under which conditions (here: speed or accuracy instructions) accuracy or RT based measures should be more informative about the underlying ANS representations. According to this model, it can be assumed that if participants prefer accuracy over speed (i.e., in the accuracy instruction condition), this will lead to longer RTs and overall fewer errors. Moreover, in case participants prefer accuracy over speed, the influence of the overlapping ANS representations on RT should be more pronounced than when participants favor speed (i.e., in the speed instruction condition). Similarly, in case participants prefer speed over accuracy (i.e., speed instruction), they should respond faster and make more errors, and the influence of the overlapping ANS representations on accuracy should be stronger than when participants prefer accuracy (i.e., accuracy instruction) [66].

To investigate whether the influence of the overlapping ANS representations on accuracy and RT data indeed depends on task instructions, we focused on the ratio effect. The ratio effect can be calculated from both accuracy and RT data. Moreover, the overlapping ANS representations are thought to produce this effect [12,16,18]. In case the ANS is reflected in accuracy and/or RT data, the ratio effect should explain variance of these dependent variables (i.e., accuracy or RT). Therefore, the part of variance explained by the ratio between the to-be-compared numerosities in a dot comparison task should indicate to what degree accuracy or RT is influenced by the underlying ANS representations. According to the diffusion model, the explained variance of accuracy/ RT by the ratio should depend on task instruction. Following the assumptions of the diffusion model regarding the influence of the overlapping ANS representations on accuracy and RT, respectively, we expected that the part of variance in RT data explained by the ratio between the to-be-compared numerosities should be larger in the accuracy instruction condition than in the combined instruction, while being smallest in the speed instruction condition. Analogously, we expected that the part of variance in accuracy data explained by the ratio should be larger in the speed instruction condition than in the combined instruction condition and be smallest in the accuracy instruction condition.

### Materials and Methods

#### Participants

Thirty-nine students (32 female, 5 left-handed, *M*_*age*_ = 23.26 years, *SD*_*age*_ = 4.15, *age range* = 19-42 years) participated in the experiment. All students provided written informed consent prior to their participation and received course credits for compensation. The experiment was approved by the local ethics committee of the Leibniz-Institut fuer Wissenmedien in Tuebingen.

#### Stimuli and procedure

The participants were given three non-symbolic dot comparison tasks with different task instructions. In all three task versions two dot sets were presented for 200ms, followed by a mask, which remained on the screen until the participant responded. Analogous to Experiment 1, participants had to indicate which of the two spatially separated dot sets contained more dots. The 405 items (including 5 practice items), response keys, and visual angle used in the three instruction conditions were identical to those in Experiment 1 and are described in detail above. However, the three instruction conditions differed regarding the degree to which speed or accuracy was emphasized in the instruction (which was presented in written form on the screen): (1) the speed instruction asked participants to respond as fast as possible, (2) the accuracy instruction asked participants to respond as accurate as possible, and (3) the combined instruction (also used in Experiment 1) asked participants to respond as quickly and accurately as possible. Participants completed all three instruction conditions. Order of instruction conditions was counterbalanced over participants as far as possible. The condition combined instruction corresponds to the design used in Experiment 1.

#### Analysis

First, a trimming procedure excluded all RTs deviating more than 3 *SD* from the individual participant’s mean in each instruction condition (approximately 1.18% of all responses). In a next step, we investigated whether our manipulation was successful and, hence, whether performance of participants differed depending on task instructions. Moreover, we examined whether task performance was influenced by the ratio between the to-be-compared numerosities and how this ratio effect interacted with task instruction. Therefore, we conducted two repeated-measures analyses of variance (ANOVA) with task instruction as within-participant factor (accuracy, speed, or combined instruction), ratio as a continuous predictor, and accuracy and RT as dependent variable, respectively, using the R package afex [95]. When sphericity assumptions were not met, we applied Greenhouse-Geisser corrections [96].

Next, we assessed the part of variance in RT and accuracy data, respectively, being explained by the ratio between the to-be-compared numerosities using *R*^*2*^ (i.e., the coefficient of determination) for RTs and the Pseudo-R^2^ $R_{LR}^{2}$ for accuracy based on log-likelihoods of the fitted and the null model (intercept only model). We calculated *R*^*2*^ and $R_{LR}^{2}$ for each participant in each instruction condition. *R*^*2*^ was calculated using a linear model with the ratio between the to-be-compared numerosities as predictor and RT as dependent variable. $R_{LR}^{2}$ was calculated with a generalized linear model with a binomial error distribution and the logit as link function using the R package MuMIn [97]. The ratio between the to-be-compared numerosities served as predictor and accuracy as dependent variable. To investigate whether the part of variance in accuracy and RT data, respectively, explained by the ratio depended on task instruction, as expected from the diffusion model, we conducted two separate repeated-measures ANOVAS, with $R^{2}\text{/}R_{LR}^{2}$ as dependent variable and task instruction as within-participant factor.

Moreover, in order to replicate the results from our first experiment and to investigate whether the relationship between ANS measures was influenced by task instruction we conducted separate correlation analyses for each instruction condition. Thus, we calculated separately for each participant and each task instruction condition the ANS measures mean accuracy, NDE_acc, NRE_acc, the Weber fractions, mean RT, NDE_RT, NRE_RT as well as the composite scores (for details on the calculation of these measures see the analysis section of Experiment 1). As in Experiment 1, we checked the reliability of all these measures (see analysis section Experiment 1 for details on the method). Moreover, we employed Spearman’s rank correlation coefficients and used the Benjamini-Hochberg procedure to correct for multiple comparisons [87,88].

### Results

#### Analyses of variance–manipulation check

**Fig 7:**
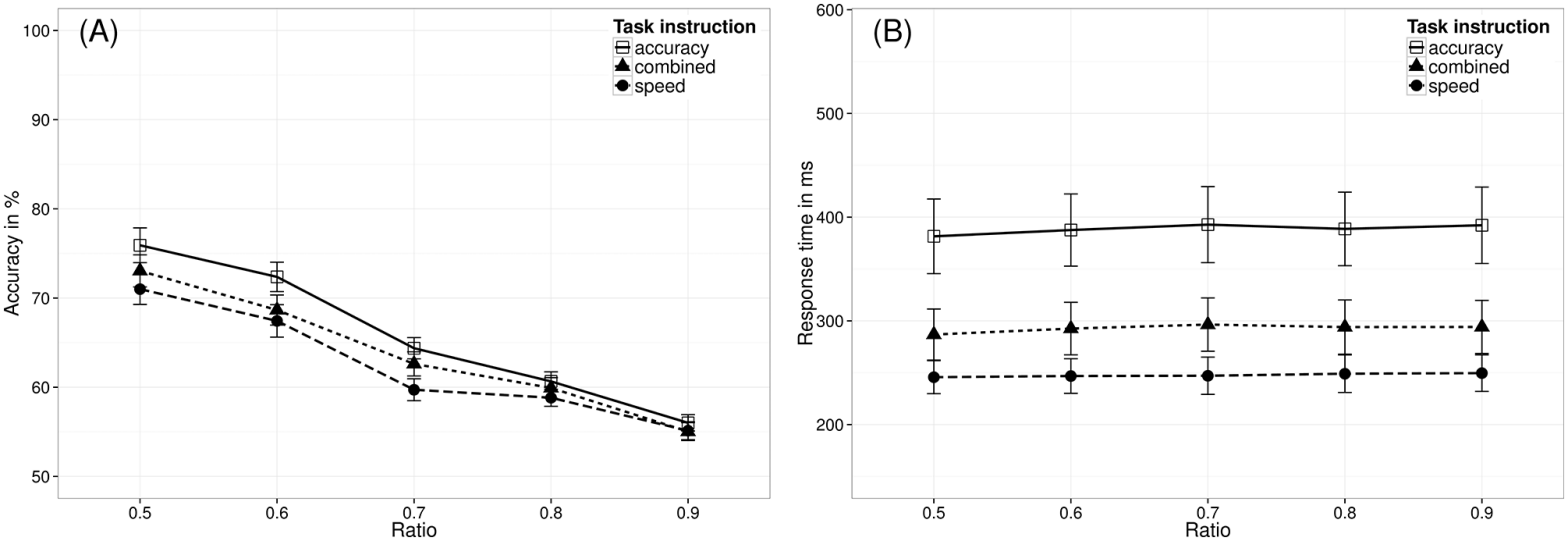
Mean accuracy (A) and mean RT (B) as a function of ratio and task instruction. Error bars represent standard errors of the mean.

Using a repeated-measures ANOVA with task instruction as within-participant factor (accuracy, speed, or combined instruction) and the ratio between the to-be-compared numerosities as continuous predictor, we investigated whether participants’ task performance was influenced by task instruction (see Fig 7). The results revealed a significant effect of task instruction both on mean accuracy, *F*(2,76) = 5.27, *p* = .007, and mean RT, *F*(2,76) = 11.15, *p* < .001. Participants performed more accurately under the accuracy instruction (*M* = 66.03%, *SD* = 6.55%) than under the combined instruction (*M* = 64.01%, *SD* = 7.40%), *t*(38) = 2.23, *p*_*corrected*_ = .048, or under the speed instruction (*M* = 62.57%, *SD* = 7.17%), *t*(38) = 3.23, *p*_*corrected*_ = .008. However, mean accuracy did not differ significantly between the combined instruction and the speed instruction, *t*(38) = 1.18, *p*_*corrected*_ = .245. Furthermore, mean RTs were significantly longer under the accuracy instruction (*M* = 388.38 ms, *SD* = 223.61 ms) than under the combined instruction (*M* = 292.68 ms, *SD* = 158.60 ms), *t*(38) = 2.70, *p*_*corrected*_ = .016, or under the speed instruction (*M* = 247.62 ms, *SD* = 107.07 ms), *t*(38) = 4.51, *p*_*corrected*_ < .001. Comparable to accuracy, mean RTs in the combined instruction and the speed instruction condition did not differ significantly, *t*(38) = 1.93, *p*_*corrected*_ = .061. Taken together, these results revealed that the different instructions induced the typical pattern of a speed-accuracy trade-off (i.e., higher mean accuracy was accompanied by longer RTs and vice versa).

Furthermore, the results of the ANOVAs also revealed a significant effect of ratio on both accuracy (*M*_*NRE_acc*_ = -4.56%), *F*(1,38) = 171.50, *p* < .001, and RT (*M*_*NRE_RT*_ = 1.68 ms), *F*(1,38) = 8.31, *p* = .006. We found the typical pattern for the ratio effects, i.e., accuracy decreased as ratio increased, whereas RT increased with ratio.

Moreover, for accuracy there was a significant interaction between task instruction and ratio, *F*(2,76) = 3.42, *p* = .038, indicating that the size of NRE_acc differed significantly between instruction conditions (see Fig 7). Therefore, we calculated the ratio effect for each participant separately for each instruction condition using a linear model with ratio as predictor and mean accuracy as dependent variable. Planned contrasts revealed that the ratio effect in the accuracy instruction condition (*M*_*NRE_acc*_ = -5.15%) was significantly larger than in the speed instruction condition (*M*_*NRE_acc*_ = -4.04%), *t*(38) = -2.75, *p*_*corrected*_ = .028. The other contrasts were not significant, *p*_*corrected*_ > .222. In contrast, there was no significant interaction between task instruction and ratio for RT, *F*(2,76) = 0.64, *p* = .531, indicating that the size of NRE_RT was equivalent for the three task instructions (see Fig 7).

#### Analyses of variance–explained variance by ratio

**Fig 8:**
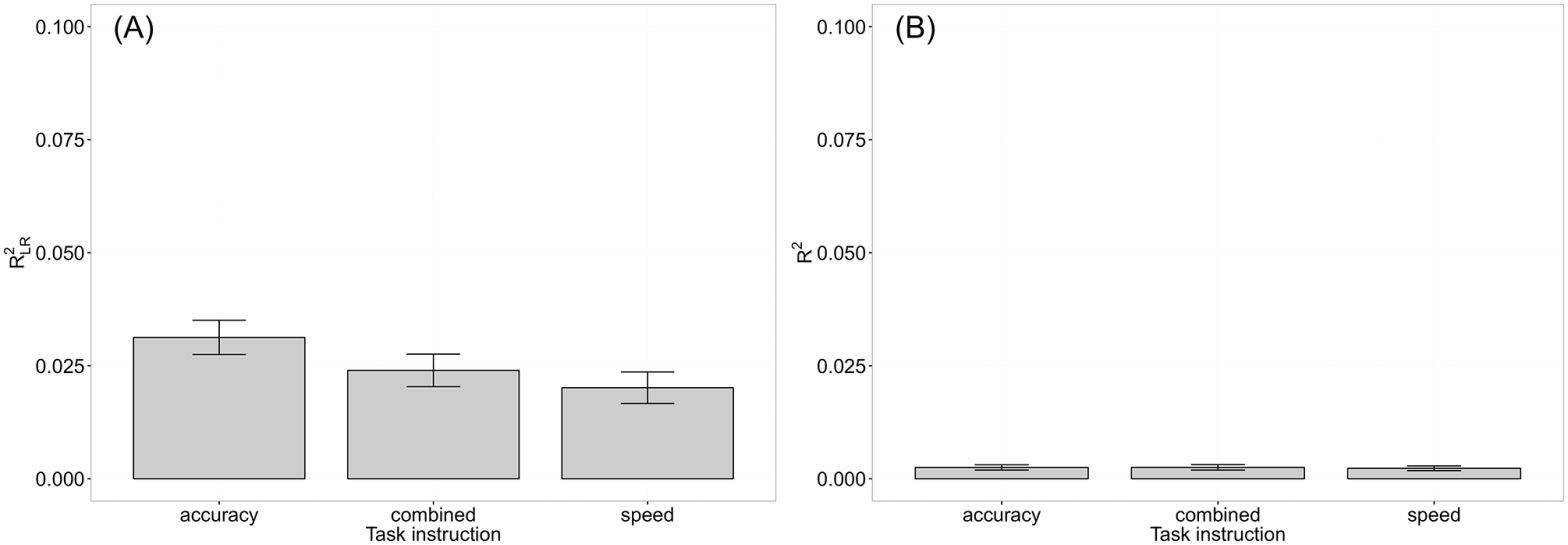
Part of variance explained by the ratio between the to-be-compared numerosities. Mean $R_{LR}^{2}$ (panel A) and mean *R*^*2*^ (panel B), indicating the part of variance in accuracy data ($R_{LR}^{2}$) or RT data (*R*^2^) explained by the ratio, separately for each task instruction condition. Error bars represent standard errors of the mean.

In a next step, we investigated whether the part of variance in accuracy and RT data, respectively, explained by the ratio was influenced by task instruction. Therefore, we conducted a repeated-measures ANOVA with task instruction as within-participant factor (accuracy, combined, or speed instruction) and $R_{LR}^{2}$ as dependent variable. We used $R_{LR}^{2}$ as an index for the part of variance in accuracy data explained by the ratio between the to-be-compared numerosities. The intercept of the ANOVA for $R_{LR}^{2}$ was significant, *F*(1,38) = 77.33, *p* < .001, indicating that the part of variance in accuracy explained by the ratio between the to-be-compared numerosities differed significantly from zero. In line with our expectations, $R_{LR}^{2}$ differed significantly between the three instruction conditions (see Fig 8), *F*(2,76) = 4.26, *p* = .018. However, planned contrasts revealed that the direction of the effect was contrary to our expectations based on the diffusion model. $R_{LR}^{2}$ was significantly larger under the accuracy instruction (mean $R_{LR}^{2}=0.031$) than under the speed instruction (mean $R_{LR}^{2}=0.020$), *t*(38) = 3.46, *p*_*corrected*_ = .004. All other planned contrasts were not significant (*p*_*corrected*_ > .134). Nevertheless, the ratio explained only between 2% and 3.1% of the variance in accuracy.

Additionally, we conducted a repeated-measures ANOVA with task instruction as within-participant factor (accuracy, combined, or speed instruction) and *R*^*2*^ as dependent variable. *R*^*2*^ indicates the part of variance in RT data explained by the ratio between the to-be-compared numerosities. The intercept of the ANOVA for *R*^*2*^ was significant, *F*(1,38) = 63.79, *p* < .001, indicating that the part of variance in RT explained by the ratio was significantly different from zero. Contrary to our expectations, *R*^*2*^ (mean *R*^*2*^ = 0.002) did not differ significantly between the three task instruction conditions (see Fig 8), *F*(2,76) = 0.04, *p* = .962. Thus, on average only 0.2% of the variance in RT was explained by the ratio.

#### Correlation analysis

Moreover, we investigated the relationship between the ANS measures separately for each instruction condition (see Tables 4–6). Split-half reliabilities for all measures (in all three instruction conditions) were in the acceptable range for group studies according to the classification of Murphy and Davidshover (all Spearman-Brown corrected reliabilities above 0.61) [89]. Overall, the correlation patterns were very similar in all instruction conditions and will be described jointly. In line with the results of our first experiment, all accuracy based measures were highly correlated. As in Experiment 1, RT based measures were not as homogenous as accuracy based measures. While NDE_RT and NRE_RT were highly related, the correlations between these two measures and mean RT were substantially smaller. Moreover, there was a large, positive correlation between mean accuracy and RTs, providing evidence for a speed-accuracy trade-off. In contrast to the results of our first experiment, the efficiency score was also strongly correlated with accuracy measures, however, it was still most strongly correlated with mean RT. As in Experiment 1, the *z*-score was equally strongly related with accuracy and RT.

**Table 4:** Spearman correlation coefficients between all ANS measures under accuracy instruction.

	(1)	(2)	(3)	(4)	(5)	(6)	(7)	(8)	(9)	(10)
(1) Mean acc	1.00									
(2) NDE_acc	.83***	1.00								
(3) NRE_acc	-.83***	-.97***	1.00							
(4) w (lin)	-.98***	-.87***	.88***	1.00						
(5) w (log)	-.98***	-.86***	.87***	1.00***	1.00					
(6) w (mod.log)	-.77***	-.76***	.80***	.80***	.78***	1.00				
(7) Mean RT	.80***	.79***	-.79***	-.80***	-.79***	-.82***	1.00			
(8) NDE_RT	-.51**	-.45**	.42**	.45**	.44**	.31	-.44**	1.00		
(9) NRE_RT	.42**	.42**	-.36*	-.34*	-.31	-.25	.34*	-.92***	1.00	
(10) Efficiency	.71***	.72***	-.72***	-.70***	-.70***	-.77***	.98***	-.40*	.30	1.00
(11) z-score	.93***	.85***	-.86***	-.94***	-.93***	-.86***	.95***	-.46**	.36*	.89***

Note.

* *p* < .05,

** *p* < .01,

*** *p* < .001;

*p*-values were adjusted for multiple testing using the Benjamini-Hochberg procedure. Cells were colored grey whenever the direction of the correlations was opposite to our predictions from Table 1. Mean acc = mean accuracy, NDE_acc = numerical distance effect calculated based on accuracy data, NRE_acc = numerical ratio effect calculated based on accuracy data, w = Weber fraction calculated from the linear model (lin), the logarithmic model (log) or the modified logarithmic model (mod.log), Mean RT = mean response time, NDE_RT = numerical distance effect calculated based on RT data, NRE_RT = numerical ratio effect calculated based on RT data, Efficiency = inverse efficiency score.

**Table 5:** Spearman correlation coefficients between all ANS measures under combined instruction.

	(1)	(2)	(3)	(4)	(5)	(6)	(7)	(8)	(9)	(10)
(1) Mean acc	1.00									
(2) NDE_acc	.75***	1.00								
(3) NRE_acc	-.77***	-.96***	1.00							
(4) w (lin)	-.96***	-.81***	.83***	1.00						
(5) w (log)	-.96***	-.82***	.83***	1.00***	1.00					
(6) w (mod.log)	-.78***	-.72***	.79***	.83***	.80***	1.00				
(7) Mean RT	.84***	.71***	-.74***	-.80***	-.78***	-.82***	1.00			
(8) NDE_RT	-.53***	-.49**	.50**	.47**	.47**	.54***	-.63***	1.00		
(9) NRE_RT	.47**	.39*	-.45**	-.38*	-.41*	-.46**	.43**	-.84***	1.00	
(10) Efficiency	.72***	.61***	-.63***	-.62***	-.63***	-.69***	.96***	-.60***	.40*	1.00
(11) z-score	.95***	.76***	-.77***	-.93***	-.92***	-.84***	.95***	-.63***	.47**	.87***

Note.

* *p* < .05,

** *p* < .01,

*** *p* < .001;

*p*-values were adjusted for multiple testing using the Benjamini-Hochberg procedure. Cells were colored grey whenever the direction of the correlations was opposite to our predictions from Table 1. Mean acc = mean accuracy, NDE_acc = numerical distance effect calculated based on accuracy data, NRE_acc = numerical ratio effect calculated based on accuracy data, w = Weber fraction calculated from the linear model (lin), the logarithmic model (log) or the modified logarithmic model (mod.log), Mean RT = mean response time, NDE_RT = numerical distance effect calculated based on RT data, NRE_RT = numerical ratio effect calculated based on RT data, Efficiency = inverse efficiency score.

**Table 6:** Spearman correlation coefficients between all ANS measures under speed instruction.

	(1)	(2)	(3)	(4)	(5)	(6)	(7)	(8)	(9)	(10)
(1) Mean acc	1.00									
(2) NDE_acc	.76***	1.00								
(3) NRE_acc	-.76***	-.97***	1.00							
(4) w (lin)	-.96***	-.81***	.80***	1.00						
(5) w (log)	-.96***	-.80***	.80***	1.00***	1.00					
(6) w (mod.log)	-.83***	-.85***	.84***	.88***	.88***	1.00				
(7) Mean RT	.81***	.74***	-.73***	-.72***	-.72***	-.73***	1.00			
(8) NDE_RT	-.50**	-.33*	.36*	.52**	.50**	.50**	-.52**	1.00		
(9) NRE_RT	.36*	.31	-.36*	-.57**	-.55**	-.50**	.34*	-.90***	1.00	
(10) Efficiency	.68***	.66***	-.64***	-.56**	-.55**	-.56***	.96***	-.52**	.33*	1.00
(11) z-score	.95***	.78***	-.77***	-.91***	-.91***	-.84***	.94***	-.52**	.37*	.84***

Note.

* *p* < .05,

** *p* < .01,

*** *p* < .001;

*p*-values were adjusted for multiple testing using the Benjamini-Hochberg procedure. Cells were colored grey whenever the direction of the correlations was opposite to our predictions from Table 1. Mean acc = mean accuracy, NDE_acc = numerical distance effect calculated based on accuracy data, NRE_acc = numerical ratio effect calculated based on accuracy data, w = Weber fraction calculated from the linear model (lin), the logarithmic model (log) or the modified logarithmic model (mod.log), Mean RT = mean response time, NDE_RT = numerical distance effect calculated based on RT data, NRE_RT = numerical ratio effect calculated based on RT data, Efficiency = inverse efficiency score.

### Discussion

In Experiment 2, we investigated under which instruction conditions accuracy and RT measures, respectively, were more informative about the underlying ANS representations. Therefore, we determined the part of variance in accuracy/ RT explained by the ratio between the to-be-compared numerosities, which indicates to which degree accuracy/ RT were influenced by the underlying ANS representations. The results contradicted our expectations based on the diffusion model: for accuracy data, the part of variance explained by ratio was larger when participants preferred accuracy over speed (i.e., accuracy instruction) than when participants favored speed (i.e., speed instruction). This pattern of results is exactly the opposite from what we expected from the diffusion model. Nevertheless, it suggests that accuracy is more informative about the underlying representation in conditions, in which participants favor accuracy than in conditions, in which they favor speed.

Also the results for RT deviated from the assumptions based on the diffusion model. We expected that the part of variance in RT data explained by the ratio of the to-be-compared numerosities should be larger under accuracy instruction than under speed instruction. However, we found that the part of variance in RT explained by ratio did not differ between the three instruction conditions indicating that the influence of the overlapping ANS representations on RT was not affected by participants’ preferences for speed or accuracy.

While we are well aware of the fact that it is inappropriate to compare *R*^*2*^ and Pseudo-*R*^*2*^, as Pseudo-*R*^*2*^ produces lower values [98], we wish to nevertheless note that in our experiment the Pseudo-*R*^*2*^ (for accuracy) was more than ten times larger than *R*^*2*^ (for RT). Considering that Pseudo-*R*^*2*^ produces smaller values than *R*^*2*^, this pattern may indicate that in the present experiment, accuracy based measures appeared to be more informative about the underlying ANS representations than RT based measures.

Regarding the interrelations of ANS measures we found a very similar pattern for all three instruction conditions: all accuracy based measures were highly related, as well as NDE_RT and NRE_RT. Moreover, there was a high positive correlation between mean accuracy and mean RT, indicating a speed-accuracy trade-off. Both the efficiency score and the *z*-score correlated highly with mean accuracy and mean RT. The consistent pattern of results suggests that the relationship between ANS measures was not influenced by the individual preference for speed or accuracy.

## Experiment 3

In our third experiment, we further investigated whether the influence of the underlying ANS representations on accuracy (as well as RT) depends on participants’ preferences for accuracy or speed. In Experiment 2, we manipulated these preferences explicitly using either speed, accuracy or combined instructions. In Experiment 3, we manipulated these preferences implicitly by varying the presentation duration of the stimuli. In particular, we used four variants of a non-symbolic dot comparison task differing only with respect to the presentation duration of the stimuli. We employed three task variants with restricted presentation duration–more precisely, we used both very short presentation durations (50ms, 200ms) and one substantially longer presentation duration (2400ms). Additionally, we used one task variant with unlimited (self-paced) presentation duration (i.e., the stimuli were presented until the participants responded).

In all task variants, participants were instructed to respond as quickly and as accurately as possible. Differences regarding presentation duration should influence the preferences for accuracy or speed, whereby smaller presentation durations (50ms or 200ms) should induce a preference for speed, whereas longer or unlimited presentation durations (2400ms or unlimited) should induce a preference for accuracy. Similarly to our second experiment, the diffusion model suggests that in conditions, in which participants prefer accuracy over speed (e.g., because the presentation duration is rather long or unlimited), the influence of the overlapping ANS representations on RT should be more pronounced than under conditions, in which participants prefer speed over accuracy (e.g., because of smaller presentation durations). Similarly, it can be assumed that under conditions, in which participants prefer speed over accuracy (i.e., for shorter presentation durations), the influence of the overlapping ANS representations on accuracy should be stronger than under conditions, in which participants prefer accuracy over speed (i.e., longer or unlimited presentation durations). Again, we focused on the part of variance being explained by the ratio as an indicator for the degree to which accuracy and RT, respectively, are influenced by the underlying ANS representations.

Based on the diffusion model, we expected that the part of variance in RT data explained by the ratio between the to-be-compared numerosities should be larger in the conditions with 2400ms or unlimited presentation duration than in the conditions with shorter presentation durations. In contrast, we expected that the part of variance in accuracy data explained by the ratio should be larger in the conditions with 50ms and 200ms presentation duration than in the condition with 2400ms or unlimited presentation duration.

### Materials and Methods

#### Participants

Fourty-six adults (35 female, 6 left-handed, *M*_*age*_ = 22.30 years, *SD*_*age*_ = 3.00, *age range* = 18-33 years) participated in the experiment. All participants provided written informed consent prior to their participation and received financial compensation of 8€ per hour. The experiment was approved by the local ethics committee of the Leibniz-Institut fuer Wissensmedien in Tuebingen.

#### Stimuli and procedure

Participants performed four different non-symbolic dot comparison tasks differing only with regard to presentation duration of the stimuli (item set, response keys, and visual angles identical in all four conditions as well as identical to Experiment 1 and 2, see above for a more detailed description). Order of presentation duration conditions was counterbalanced across participants. In one condition, stimulus presentation duration was unlimited, thus the two to-be-compared dot sets were presented until participants responded. In the other three conditions, stimuli were displayed for 50ms, 200ms or 2400ms, respectively. After this respective period the dot sets were replaced by a mask, which remained on the screen until participants responded. Participants were instructed to indicate as quickly and accurately as possible, which of the two presented dot sets contained more dots. The task variant employing presentation duration of 200ms corresponds to the design of Experiment 1 and the condition *combined instruction* in Experiment 2.

#### Analysis

Again, we first ran a trimming procedure excluding all RTs deviating more than 3 *SD* from the individual participant’s mean in each presentation duration condition (approximately 1.74% of all responses). For all conditions with restricted presentation durations (i.e., 50ms, 200ms, and 2400ms) RT was defined as the period of time between the end of stimulus presentation and the participant’s response. However, for the unlimited presentation duration condition RT was defined as the time between the onset of stimulus presentation and participant’s response.

Next, we examined whether (1) performance of the participants differed depending on presentation duration, (2) task performance was influenced by the ratio between the to-be-compared numerosities, and (3) how this ratio effect interacts with presentation duration. Therefore, we conducted two repeated-measures ANOVAs with presentation duration as within-participant factor (50ms, 200ms, 2400ms, and unlimited), ratio as continuous predictor, and accuracy and RT as dependent variable, respectively. In a next step, we investigated, analogous to Experiment 2, whether the part of variance in accuracy and RT data, respectively, explained by the ratio depended on presentation duration. Therefore, we conducted two separate repeated-measures ANOVAs, with $R^{2}/R_{LR}^{2}$ as dependent variable and presentation duration as within-participant factor (for details on the calculation of $R^{2}/R_{LR}^{2}$ see the analysis section of Experiment 2). Moreover, Greenhouse-Geisser corrections were used when the sphericity assumption was not met [96].

Furthermore, we studied whether the relationship between the ANS measures was influenced by presentation duration, using four separate correlation analyses. We calculated Spearman’s rank correlation coefficients between the following ANS measures: mean accuracy, NDE_acc, NRE_acc, the Weber fractions, mean RT, NDE_RT, NRE_RT, the inverse efficiency score and the *z*-score (for details on the calculation of these measures see the analysis section of Experiment 1). As in previous studies, we checked reliabilities of the measures used and corrected the respective *p*-values for multiple comparisons, using the Benjamini-Hochberg procedure [88].

### Results

#### Analyses of variance–manipulation check

**Fig 9:**
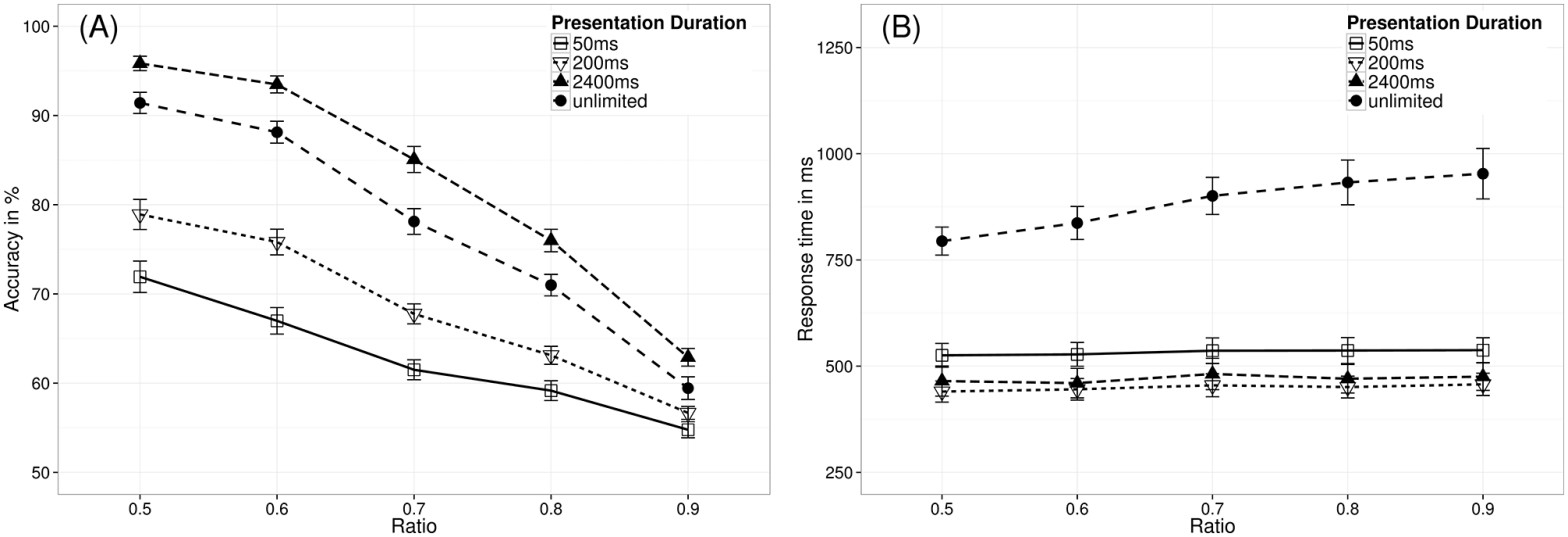
Mean accuracy (A) and mean RT (B) as a function of ratio and presentation duration. Error bars represent standard errors of the mean.

We conducted two repeated-measures ANOVAs with presentation duration as within-participant factor (50ms, 200ms, 2400ms, and unlimited), the ratio between the to-be-compared numerosities as continuous predictor, and accuracy and RT as dependent variable, respectively. Results revealed a significant effect of presentation duration on both mean accuracy, *F*(3,135) = 193.40, *p* < .001, and mean RT, *F*(3,135) = 53.16, *p* < .001. It became evident that for the restricted presentation durations, participants performed more accurately, the longer the presentation duration got. Moreover, in the unlimited presentation duration condition participants were more accurate than in the 200ms condition but less accurate than in the 2400ms conditions (see Fig 9, all *p*_*corrected*_ < .001). For RTs a divergent pattern was observed. Participants responded significantly slower in the unlimited condition than in all conditions with restricted presentation durations (see Fig 9; all *p*_*corrected*_ < .001). Moreover, participants were significantly slower in the 50ms condition than in the 200ms condition, *t*(45) = -2.95, *p* = .008. All other pairwise comparisons were not significant (all *p*_*corrected*_ > .18).

Furthermore, we replicated the ratio effect for accuracy (*M*_*NRE_acc*_ = -6.59%), *F*(1,45) = 865.20, *p* < .001, as well as for RT (*M*_*NRE_RT*_ = 13.33 ms), *F*(1,45) = 29.72, *p* < .001, i.e., accuracy decreased as ratio increased, whereas RT increased with ratio.

In addition, there was a significant interaction between presentation duration and ratio, for both accuracy, *F*(3,135) = 56.45, *p* < .001, and RT, *F*(3,135) = 19.15, *p* < .001 (Greenhouse-Geisser coefficient (GG): .39) indicating that the size of the ratio effects differed significantly between the conditions (see Fig 9). For accuracy, the ratio effect was largest for the conditions 2400ms and unlimited (2400: NRE_acc = -8.33%, *t*(45) = -39.02, *p* < .001; unlimited: NRE_acc = -8.11%, *t*(45) = -30.61, *p* < .001; all *t*-tests were compared against zero). The ratio effects in these two conditions were significantly larger than the ratio effect in the 200ms conditions (NRE_acc = -5.72%, *t*(45) = -14.77, *p* < .001), which in turn was larger than the ratio effect for the 50ms condition (NRE_acc = -4.21%, *t*(45) = -11.10, *p* < .001). Planned contrasts revealed that all pairwise comparisons (except the comparison of NRE_acc in the 2400ms condition and the unlimited condition, *t*(45) = -0.78, *p* = .430) were significant (all *p*_*corrected*_ < .001).

For RTs the mean ratio effect in the unlimited condition (NRE_RT = 42.52 ms, *t*(45) = 4.88, *p* < .001) was significantly larger than the ratio effects in all conditions with restricted presentation duration (50ms: NRE_RT = 3.43 ms, *t*(45) = 2.56, *p* = .013; 200ms: NRE_RT = 4.20 ms, *t*(45) = 3.33, *p* = .002; 2400ms: NRE_RT = 3.18 ms, *t*(45) = 1.58, *p* = .120; pairwise comparisons: all *p*_*corrected*_ < .001). All other pairwise comparisons were not significant (all *p*_*corrected*_ > .809).

#### Analyses of variance–explained variance by ratio

We further investigated whether the part of variance in accuracy and RT data, respectively, explained by the ratio between the to-be-compared numerosities was influenced by presentation duration. Therefore, we conducted two repeated-measures ANOVAs with presentation duration as within-participant factor (50ms, 200ms, 2400ms, and unlimited) and $R^{2}/R_{LR}^{2}$ as dependent variable, respectively. Again, *R*^*2*^ was used as an index for the part of variance in RT data explained by the ratio between the to-be-compared numerosities, whereas $R_{LR}^{2}$ was used an index for the part of variance in accuracy data explained by the ratio.

**Fig 10:**
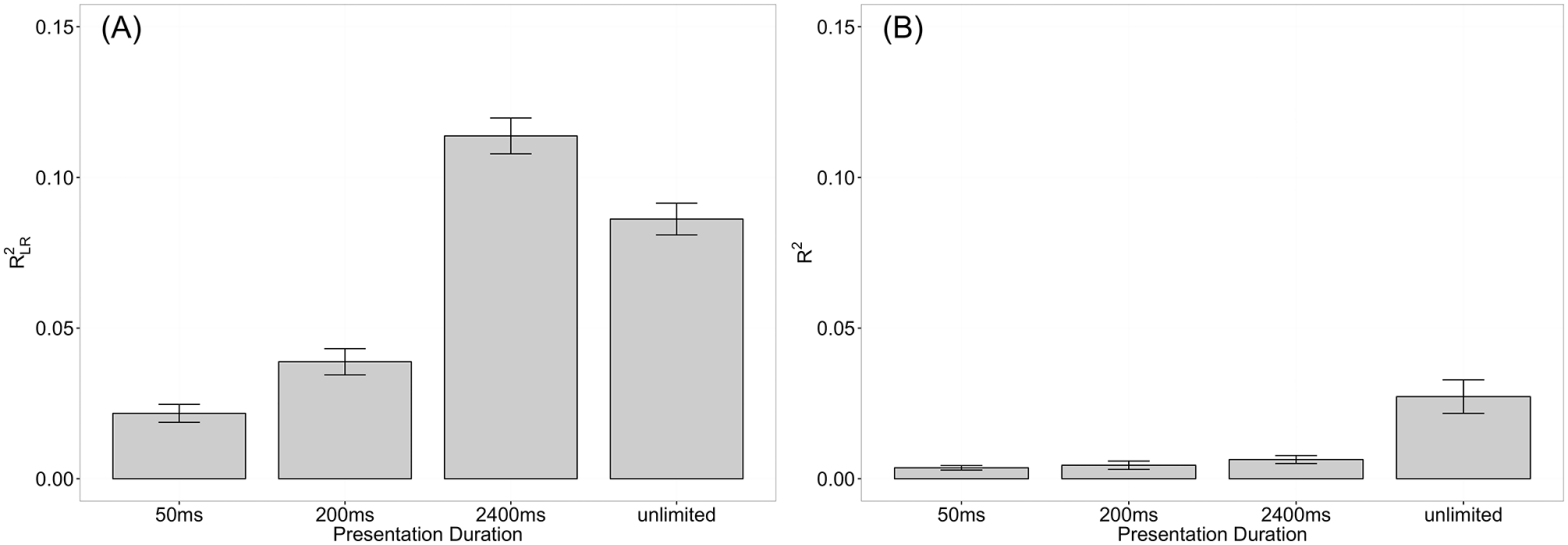
Part of variance explained by the ratio between the to-be-compared numerosities. Mean $R_{LR}^{2}$ (panel A) and mean *R*^*2*^ (panel B), indicating the part of variance in accuracy data ($R_{LR}^{2}$) or RT data (*R*^*2*^) explained by the ratio, separately for each presentation duration condition. Error bars represent standard errors of the mean.

Our results revealed that the part of variance in accuracy explained by the ratio (i.e., mean $R_{LR}^{2}$) differed significantly from zero, *F*(1,45) = 320.75, *p* < .001. Moreover, $R_{LR}^{2}$ differed significantly between the four presentation duration conditions, *F*(3,135) = 141.79, *p* < .001 (GG: .82). However, the direction of the effect contradicted our expectations (see Fig 10, all *p*_*corrected*_ < .001). For restricted presentation duration conditions, the part of variance in accuracy explained by the ratio increased with presentation duration. For the unlimited condition, the part of variance explained by the ratio was smaller than in the 2400ms condition, but larger than in the 200ms condition.

Furthermore, also the part of variance in RT explained by the ratio (i.e., mean *R*^*2*^) was significantly different from zero, *F*(1,45) = 44.04, *p* < .001. In addition, *R*^*2*^ differed significantly between the four presentation duration conditions, *F*(3,135) = 15.05, *p* < .001 (GG: .41). *R*^*2*^ in the unlimited condition was significantly larger than *R*^*2*^ of each condition with restricted presentation duration (see Fig 10; all *p*_*corrected*_ < .001). All other pairwise comparisons were not significant (all *p*_*corrected*_ > .138).

#### Correlation analysis

Again, we investigated the relationship between the ANS measures separately for each of the four presentation duration conditions (see Tables 7–10). In advance, we checked the split-half reliabilities for all measures (in all four conditions) and found that reliabilities for all measures were in the acceptable range for group studies according to Murphy and Davidshover (all Spearman-Brown corrected reliabilities above 0.73) [89]. Consistent with the results of our previous experiments, we observed that all accuracy based measures were strongly correlated for all presentation duration conditions. Moreover, we found that in all conditions NDE_RT and NRE_RT were highly related. However, the relationship of these two measures and mean RT differed strongly between different presentation duration conditions. In the 50ms and the 200ms condition, the correlations between NDE_RT, NRE_RT, and mean RT were only small to moderate [59], whereas in the 2400ms conditions NDE_RT/ NRE_RT and mean RT did not correlate significantly. In contrast, in the unlimited condition there was a high correlation between NDE_RT/ NRE_RT and mean RT [59]. Interestingly, also the correlation between mean accuracy and mean RT differed depending on presentation duration. For the conditions 50ms, 200ms, and unlimited, we found–in line with the findings of our previous experiments–a high, positive correlation between mean accuracy and mean RT. However, for the 2400ms condition, only a low positive correlation between these measures was observed [59]. Similar to the results of our previous experiments, the efficiency score was highly related with mean RT, whereas the correlation between the efficiency score and mean accuracy was only small or moderate. In contrast, the *z*-score was strongly related with both mean accuracy and mean RT. This pattern of results is similar to the findings from our previous experiments.

**Table 7:** Spearman correlation coefficients between all ANS measures in the 50ms condition.

	(1)	(2)	(3)	(4)	(5)	(6)	(7)	(8)	(9)	(10)
(1) Mean acc	1.00									
(2) NDE_acc	.79***	1.00								
(3) NRE_acc	-.80***	-.96***	1.00							
(4) w (lin)	-.97***	-.83***	.84***	1.00						
(5) w (log)	-.98***	-.82***	.84***	1.00***	1.00					
(6) w (mod.log)	-.80***	-.63***	.66***	.78***	.76***	1.00				
(7) Mean RT	.65***	.57***	-.57***	-.52**	-.51**	-.64***	1.00			
(8) NDE_RT	-.29	-.15	.14	.23	.24	.14	-.36*	1.00		
(9) NRE_RT	.29	.18	-.16	-.25	-.26	-.11	.26	-.91***	1.00	
(10) Efficiency	.39*	.39*	-.39*	-.25	-.24	-.44**	.93***	-.33*	.22	1.00
(11) z-score	.87***	.76***	-.76***	-.83***	-.83***	-.80***	.91***	-.33*	.28	.74***

Note.

* *p* < .05,

** *p* < .01,

*** *p* < .001;

*p*-values were adjusted for multiple testing using the Benjamini-Hochberg procedure. Cells were colored grey whenever the direction of the correlations was opposite to our predictions from Table 1. Mean acc = mean accuracy, NDE_acc = numerical distance effect calculated based on accuracy data, NRE_acc = numerical ratio effect calculated based on accuracy data, w = Weber fraction calculated from the linear model (lin), the logarithmic model (log) or the modified logarithmic model (mod.log), Mean RT = mean response time, NDE_RT = numerical distance effect calculated based on RT data, NRE_RT = numerical ratio effect calculated based on RT data, Efficiency = inverse efficiency score.

**Table 8:** Spearman correlation coefficients between all ANS measures in the 200ms condition.

	(1)	(2)	(3)	(4)	(5)	(6)	(7)	(8)	(9)	(10)
(1) Mean acc	1.00									
(2) NDE_acc	.80***	1.00								
(3) NRE_acc	-.84***	-.95***	1.00							
(4) w (lin)	-.98***	-.85***	.90***	1.00						
(5) w (log)	-.98***	-.85***	.90***	1.00***	1.00					
(6) w (mod.log)	-.61***	-.54***	.64***	.66***	.65***	1.00				
(7) Mean RT	.59***	.60***	-.59***	-.59***	-.59***	-.68***	1.00			
(8) NDE_RT	-.46**	-.41**	.34*	.40**	.41**	.12	-.31*	1.00		
(9) NRE_RT	.49**	.48**	-.44**	-.47**	-.48**	-.10	.24	-.85***	1.00	
(10) Efficiency	.32*	.39*	-.37*	-.33*	-.33*	-.57***	.93***	-.18	.07	1.00
(11) z-score	.85***	.77***	-.78***	-.85***	-.85***	-.71***	.90***	-.42**	.39*	.72***

Note.

* *p* < .05,

** *p* < .01,

*** *p* < .001;

*p*-values were adjusted for multiple testing using the Benjamini-Hochberg procedure. Cells were colored grey whenever the direction of the correlations was opposite to our predictions from Table 1. Mean acc = mean accuracy, NDE_acc = numerical distance effect calculated based on accuracy data, NRE_acc = numerical ratio effect calculated based on accuracy data, w = Weber fraction calculated from the linear model (lin), the logarithmic model (log) or the modified logarithmic model (mod.log), Mean RT = mean response time, NDE_RT = numerical distance effect calculated based on RT data, NRE_RT = numerical ratio effect calculated based on RT data, Efficiency = inverse efficiency score.

**Table 9:** Spearman correlation coefficients between all ANS measures in the 2400ms condition.

	(1)	(2)	(3)	(4)	(5)	(6)	(7)	(8)	(9)	(10)
(1) Mean acc	1.00									
(2) NDE_acc	.87***	1.00								
(3) NRE_acc	-.83***	-.98***	1.00							
(4) w (lin)	-.98***	-.93***	.91***	1.00						
(5) w (log)	-.98***	-.94***	.91***	1.00***	1.00					
(6) w (mod.log)	-.53***	-.60***	.63***	.60***	.60***	1.00				
(7) Mean RT	.11	.10	-.05	-.07	-.08	.18	1.00			
(8) NDE_RT	-.22	-.23	.24	.20	.21	-.07	.09	1.00		
(9) NRE_RT	.17	.18	-.19	-.15	-.15	.00	-.10	-.90***	1.00	
(10) Efficiency	-.10	-.08	.12	.12	.12	.30	.96***	.09	-.11	1.00
(11) z-score	.69***	.56***	-.51***	-.65***	-.65***	-.19	.70***	.02	-.04	.53***

Note.

*** *p* < .001;

*p*-values were adjusted for multiple testing using the Benjamini-Hochberg procedure. Cells were colored grey whenever the direction of the correlations was opposite to our predictions from Table 1. Mean acc = mean accuracy, NDE_acc = numerical distance effect calculated based on accuracy data, NRE_acc = numerical ratio effect calculated based on accuracy data, w = Weber fraction calculated from the linear model (lin), the logarithmic model (log) or the modified logarithmic model (mod.log), Mean RT = mean response time, NDE_RT = numerical distance effect calculated based on RT data, NRE_RT = numerical ratio effect calculated based on RT data, Efficiency = inverse efficiency score.

**Table 10:** Spearman correlation coefficients between all ANS measures in the unlimited condition.

	(1)	(2)	(3)	(4)	(5)	(6)	(7)	(8)	(9)	(10)
(1) Mean acc	1.00									
(2) NDE_acc	.85***	1.00								
(3) NRE_acc	-.81***	-.94***	1.00							
(4) w (lin)	-.98***	-.91***	.89***	1.00						
(5) w (log)	-.98***	-.91***	.89***	1.00***	1.00					
(6) w (mod.log)	-.62***	-.69***	.82***	.70***	.70***	1.00				
(7) Mean RT	.65***	.56***	-.58***	-.67***	-.67***	-.57***	1.00			
(8) NDE_RT	-.67***	-.60***	.61***	.68***	.68***	.52***	-.79***	1.00		
(9) NRE_RT	.73***	.68***	-.70***	-.75***	-.75***	-.58***	.72***	-.96***	1.00	
(10) Efficiency	.39**	.32*	-.34*	-.41**	-.42**	-.39**	.93***	-.67***	.58***	1.00
(11) z-score	.91***	.78***	-.76***	-.91***	-.91***	-.64***	.88***	-.81***	.80***	.70***

Note.

* *p* < .05,

** *p* < .01,

*** *p* < .001;

*p*-values were adjusted for multiple testing using the Benjamini-Hochberg procedure. Cells were colored grey whenever the direction of the correlations was opposite to our predictions from Table 1. Mean acc = mean accuracy, NDE_acc = numerical distance effect calculated based on accuracy data, NRE_acc = numerical ratio effect calculated based on accuracy data, w = Weber fraction calculated from the linear model (lin), the logarithmic model (log) or the modified logarithmic model (mod.log), Mean RT = mean response time, NDE_RT = numerical distance effect calculated based on RT data, NRE_RT = numerical ratio effect calculated based on RT data, Efficiency = inverse efficiency score.

### Discussion

In this third experiment, we examined under which conditions (here: varying presentation durations) accuracy or RT based measures were more informative about the underlying ANS representations. The part of variance in accuracy and RT, respectively, explained by the ratio between the to-be-compared numerosities served as an indicator of the degree to which accuracy and RT were influenced by the underlying ANS representations. Similar to Experiment 2, the results contradicted our expectations based on the diffusion model.

For accuracy data, the part of variance explained by the ratio was significantly larger in the unlimited and the 2400ms condition (i.e., for longer presentation durations) than in the 50ms or the 200ms conditions (i.e., for shorter presentation durations). This pattern of results is exactly the opposite of what we expected (i.e., a larger amount of explained variance for the 50ms and 200ms conditions than for the 2400ms and unlimited condition). According to these results, accuracy appeared to be more informative about the underlying representation in conditions, in which participants rather favor accuracy over speed (i.e., for longer presentation durations) than in conditions, in which participants favor speed over accuracy (i.e., for shorter presentation durations). Interestingly, this pattern of results is consistent with our findings from Experiment 2.

Moreover, also for RT data our results were not perfectly in line with our assumptions based on the diffusion model. We expected that the part of variance in RT data explained by the ratio should be larger in the 2400ms and unlimited conditions than in the 50ms and 200ms conditions. In line with this hypothesis we found a significantly larger part of variance in RT explained by the ratio in the unlimited condition. However, for the 2400ms condition, a divergent pattern was found, as the amount of explained variance of the 2400ms condition did not differ significantly from the two conditions with smaller presentation durations. As in Experiment 2, the part of variance explained by the ratio was substantially larger for accuracy data than for RT data, even though one cannot compare *R*^*2*^ and Pseudo-*R*^*2*^ directly [98]. Nevertheless, this pattern may provide evidence for the notion that accuracy based measures are more informative about the underlying ANS representations than RT measures.

In addition, we examined whether the relationship between the ANS measures was influenced by presentation durations. We consistently found that (1) all accuracy based measures were strongly related, (2) NDE_RT and NRE_RT were highly correlated, (3) there was a high correlation between the efficiency score and mean RT, whereas the efficiency score correlated only lowly or moderately with mean accuracy, and (4) that the *z*-score was equally strong correlated with both mean accuracy and mean RT. This pattern was stable over all conditions and, thus, unaffected by presentation durations. However, the strength of the correlations between mean RT and the other ANS measures was influenced by presentation duration. In the 2400ms presentation duration condition RT based measures were unrelated or only slightly related to accuracy based measures. Moreover, mean RT not even correlated with NDE_RT or NRE_RT. In contrast, for all other conditions, mainly medium to high correlations between these measures were found. A possible explanation for these findings might be that in the 2400ms condition, participants had a comparatively long time period to decide and prepare their response, which might entail that RT is a less valid indicator in this condition. This might also explain why the ratio effect for RT in the 2400ms condition is rather small (and not significantly different from zero).

## General Discussion

The present series of experiments aimed at systematically investigating the relationship between several indices commonly employed to assess ANS acuity. We focused both on accuracy and RT based measures as well as on composite scores combining both accuracy and RT. All these measures have been assumed to assess ANS acuity similarly and, therefore, have been used interchangeably (see e.g., [45,48]). However, recent studies reporting only low to moderate correlations between certain measures (i.e., correlations between NRE_acc/ NRE_RT and mean accuracy/ Weber fraction) questioned this assumption. Because reliability for NRE_acc and NRE_RT in previous studies was rather low (according to [89], see [39,40], but see [31]), the size of correlations among these measures might have been reduced artificially by poor reliability [60]. In order to rule out this confound, we investigated the interrelations between the measures aiming at sufficient reliability by employing a large number of trials.

In the following, we will first discuss the results for accuracy based, RT based, and composite measured separately, before elaborating on their interrelations. Subsequently, we will discuss how methodological aspects in calculating the NRE_acc can affect the interrelation of the measures. We end with a discussion of the implications of our results for research on the association of ANS acuity and math performance.

### Accuracy based measures

Consistent over all three experiments and independent from task instruction or presentation duration, all accuracy based ANS measures were highly correlated. This pattern of results was to be expected from a mathematical point of view and is in line with the assumption that all these measures assess the same underlying concept. Thus, the results of the present experiments suggest that all accuracy based measures can be used interchangeably. However, our findings are in contrast to previous studies reporting only weak or moderate correlations between NRE_acc and mean accuracy/ the Weber fraction. These inconsistencies may be explained by differences regarding the reliability of the measures; previous studies reported poor reliabilities, especially for NRE_acc [39,40]. In contrast, in the present experiments all accuracy based ANS measures were highly reliable (all Spearman-Brown corrected reliabilities above 0.83), which supports the notion that poor reliabilities may have reduced the expected correlations between accuracy based measures artificially in previous studies (see also [46]). Thus, if researchers want to make the assumption that different accuracy based measures are equivalent, they need to ensure sufficient reliability and, hence, use a very large number of trials (>400). Moreover, our findings further demonstrated the need for reliable measures, because unreliable measures reduce the potential size for the correlation between two variables [60]. If reliability is not considered, the resulting null effects might erroneously be interpreted as a lack of relationship between two between two ANS measures. This can have far-reaching consequences for theoretical assumptions or models derived from results with unreliable measures [99]. Moreover, unreliable measures lead to a situation where no clear statement regarding a correlation can be made, as the null effect can either be due to a lack of reliability or due to a missing true correlation. Thus, we propose to employ enough trials to ensure sufficiently high reliability of the measures. In our experiments, 400 trials were employed (following [39]) resulting in at least acceptable reliability at the group level.

### RT based measures

For RT based measures we did not find a pattern as homogenous and stable as for accuracy based measures. More specifically, RT based measures cannot be subsumed in one category. As expected from a mathematical point of view, NDE_RT and NRE_RT were highly correlated in all three experiments and independent of task instruction and presentation duration. In contrast, however, the size of the correlation between these measures and mean RT was substantially smaller than the correlation between NDE_RT and NRE_RT and differed considerably depending on task instruction and presentation duration. For longer presentation durations (i.e., 2400ms), these measures were even unrelated with mean RT. Thus, these measures cannot be used interchangeably. Importantly, our findings cannot be explained by poor reliabilities, as for all RT based measures Spearman-Brown corrected split-half reliabilities were in the acceptable range for group studies (all above 0.61, [89]). Moreover, in the 2400ms condition (i.e., the condition with the smallest correlations between NDE_RT/ NRE_RT and mean RT), reliabilities were even higher (all split-half reliabilities above 0.74). Hence, results based on RT based measures should be interpreted with caution and checked for a speed-accuracy trade-off (see below for a more detailed discussion).

### Composite measures

Besides accuracy and RT based measures also the inverse efficiency score was used as a measure of ANS acuity [36,43,77]. As this measure combines both accuracy and RT, it should reflect both accuracy and speed of performance and was proposed to control for a speed-accuracy trade-off [77,78] (but see [79]). However, our results revealed that the correlation between the inverse efficiency score and mean RT was substantially larger than the correlation between the inverse efficiency score and mean accuracy. Moreover, the size of the correlation between the efficiency score and mean accuracy was variable across experiments. Thus, the inverse efficiency score appeared to mainly capture RT variance and, therefore, seems to be rather inadequate as a composite score. This pattern of results may result from calculating the inverse efficiency score by dividing mean RT of correct responses by the proportion of correct responses, which weights accuracy in a non-linear manner. In order to get a composite score, which equally captures the variance of accuracy and RT, we alternatively calculated the *z*-score (by independently *z*- transforming mean accuracy and mean RT and afterwards averaging these values). Across all experiments and conditions, this alternative composite score was highly correlated both with mean accuracy and mean RT and, thus, indeed reflected accuracy *and* speed of performance. Therefore, this *z*-score composite might constitute a measure which controls for speed-accuracy trade-offs. Moreover, this score might even be used interchangeably with accuracy based measures and with mean RT.

### Interrelations between accuracy and RT based measures

The main concern of this series of experiments was to investigate whether accuracy and RT based measures correspond in assessing ANS acuity. This is of specific importance, because different accuracy and RT based measures have often been used interchangeably [40,45,46], which implicitly assumes that these measures do assess the same construct to the same extent.

First evidence against the common assumption that accuracy and RT based measures can be used interchangeably comes from studies reporting only small to moderate correlations between accuracy and RT based measures (i.e., NRE_RT [31,39,40]). However, as the reliability of NRE_RT was quite poor in two of the studies [39,40], reduced correlations between accuracy based measures and NRE_RT might be due to insufficient reliability [46,60]. In order to clarify whether the reported correlations reflect the “true” relationship between the measures or whether these correlations where artificially reduced due to poor reliability, we evaluated the relationship between accuracy and RT based measures ensuring sufficient reliability. The correlation between NRE_acc and NRE_RT was substantially larger in the present experiments than in previous studies [39,40], which might be attributed to better reliabilities (all Spearman-Brown corrected split-half reliabilities above 0.61). However, despite these better reliabilities the correlations between NRE_RT and the accuracy based measures Weber fraction and mean accuracy were comparable to the results from previous studies [31,39,40]–at least in all conditions employing a presentation duration of 200ms; for the other conditions the size of the correlations between NRE_RT and the accuracy based measures varied considerably depending on the presentation duration. This pattern of results indicates that these correlations were not artificially reduced by poorer reliabilities. Instead these results support the interpretation that these measures are indeed only lowly to moderately related. In turn, this raises the question whether these measures can indeed be used interchangeably.

Most importantly, however, there was a large, positive correlation between mean accuracy and mean RT, which we observed consistently across all experiments. There was only one exception: in the 2400ms condition of Experiment 3 these measures were almost unrelated (*r*_*s*_ = 0.11). Taken together, this pattern of results clearly contradicts the commonly held assumption that accuracy and RT based measures correspond in assessing ANS acuity, because in this case both measures should be negatively related (i.e., a more accurate ANS should be reflected in both large mean accuracy and short mean RT). Instead, the large positive correlations between mean accuracy and mean RT indicated a speed-accuracy trade-off, which provides a situation where accuracy and RT measures result in opposing conclusions regarding individual ANS acuity: higher mean accuracy is interpreted as reflecting a more accurate ANS, however, this is associated with longer mean RTs, which are usually interpreted as an index for a less accurate ANS acuity. Therefore, it is impossible to make unambiguous conclusion regarding the ANS acuity of a participant and it is obvious that these measures cannot be employed interchangeably.

Importantly, these findings directly raise the question which measure should be used to characterize the underlying ANS representations in situations where accuracy and RT measures do not correspond in assessing ANS acuity. In other words: which measure should be used, if accuracy and RT are inversely related (as in the case of a speed-accuracy trade-off) or if both measures are unrelated (see 2400ms condition, Experiment 3)?

To investigate which measure (accuracy or RT) was more informative about the underlying ANS representations (depending on an individual’s preference for speed or accuracy), we considered the diffusion model [66,75]. According to the diffusion model the influence of ANS representations on accuracy and RT, respectively, differs depending on participants’ preferences for either accuracy or speed. We used two approaches to manipulate participants’ preferences for accuracy or speed to evaluate these assumptions. In Experiment 2, we employed three different instruction conditions which emphasized either accuracy, speed, or both accuracy and speed to explicitly manipulate participants’ preferences for accuracy or speed. In Experiment 3, we used a more implicit approach to manipulate the preference for accuracy or speed by varying the presentation duration of the stimuli.

Based on the diffusion model, we expected that when participants prefer accuracy over speed (which results in overall high accuracy), the influence of overlapping ANS representations on RT should be more pronounced than when participants favor speed. Similarly, in case participants prefer speed over accuracy (which results in overall very fast responses), the influence of overlapping ANS representations on accuracy should be stronger than when participants prefer accuracy [66]. Participants should prefer accuracy over speed in the accuracy instruction condition (i.e., when accuracy is explicitly instructed in Experiment 2) and in the conditions with long (i.e., 2400ms and unlimited) presentation duration (Experiment 3). Conversely, participants should prefer speed over accuracy in the speed instruction condition (i.e., when speed is explicitly instructed in Experiment 2) and in the conditions with short presentation durations (50ms, 200ms in Experiment 3).

We checked our manipulations and found—in line with our expectations—that participants were indeed more accurate in those conditions, in which they were supposed to prefer accuracy over speed and responded faster in conditions, in which they were supposed to prefer speed over accuracy (with the only exception of the 2400ms condition: here mean RT did not differ from the other restricted presentation duration conditions). However, both the results from Experiment 2 and 3 contradicted the assumptions based on the diffusion model. Instead our results consistently revealed that the influence of ANS representations on accuracy was significantly stronger when participants favored accuracy over speed (i.e., in the condition accuracy was explicitly instructed as well as in the conditions with 2400ms and unlimited presentation duration). This pattern of results is exactly the opposite from what we expected based on the diffusion model. Moreover, the influence of ANS representations on RT was largely unaffected from individuals’ preferences for accuracy or speed, which again contradicts the assumptions based on the diffusion model. Furthermore, the part of variance in RT explained by the ratio between the to-be-compared numerosities (as an indicator for the effect of ANS representations) was substantially smaller than the part of variance in accuracy explained by the ratio. This pattern of results might indicate that accuracy measures may generally be more informative about the underlying ANS representations than RT measures and should, therefore, be preferred as measures of ANS acuity.

This deviation of the present results from expectations based on the diffusion model might indicate that theoretical assumptions and requirements for the diffusion model have not been met. For instance, it might be problematic that the diffusion model was developed to account for single-stage decisions [100], because previous studies provided first evidence suggesting the involvement of more complex processes (and maybe more than one processing stage) in non-symbolic dot comparison. In particular, the influence of visual solution strategies was discussed [56–58]. Moreover, depending on the congruency of visual properties, inhibitory control is required [57,91,101]. Additionally, participants might double-check their solutions using different strategies [100], especially in those conditions in which they favor accuracy. In conditions, in which participants have more time to decide (i.e., long or unlimited presentation duration), they might also use several steps to come to their decision, e.g., (1) estimate the number of dots in the left set and transfer it to a symbolic number [102], (2) estimate the number of dots in the right set and transfer it to a symbolic number, and (3) perform symbolic number comparison involving inhibitory control processes [103–106]). In case participants refer to such strategies, the solution of a dot comparison task is no longer a single-decision process, which questions the suitability of a simple diffusion model. Also in our experiments there was an influence of visual properties on task performance, indicating the involvement of visual strategies. In particular, the visual parameters in the modified logarithmic model had significantly influence on task performance (e.g., for Experiment 1, log size: *χ²* (1) = 61.59, *p* < .001; log spacing *χ²*(1) = 80.24, *p* < .001).

The involvement of additional processes challenges the validity of ANS measures [40,46,56,58,101], because these indices do no longer reflect ANS acuity uniquely, but are also influenced by domain-general abilities like inhibitory control or the processing of visual properties of the stimuli. A first approach to assess ANS acuity independently of the effects of visual properties of the stimuli was proposed by DeWind and colleagues [55]. These authors added two parameters to the logarithmic model capturing the effects of non-numerical, visual properties of the stimuli. This modification of the logarithmic model is capable to separate ANS acuity from effects of visual stimulus properties. Therefore, the estimates of ANS acuity based on this model should be more theoretically valid [55] than the other accuracy based measures.

However, not only visual properties of the stimuli affect task performance and, hence, measures of ANS acuity. There are various other design parameters which may influence task performance, and, thus, might bias the resulting estimates of ANS acuity. For example, presentation duration was found to influence task performance and the Weber fraction, whereby task performance increases (i.e., the Weber fraction decreases) as presentation duration increases [107]. In Experiment 3 we replicated this finding. Moreover, also characteristics of the item set, like set size [101] or the ratios between the to-be-compared numerosities [40], were observed to influence task performance/ the Weber fraction. Furthermore, previous studies differed regarding the arrangement of the dot sets (i.e., spatially separated, sequentially, or intermixed) as well as the color and/or shapes of the stimuli. In this context, Price and colleagues (2012) found that ANS measures derived from different arrangement conditions differed significantly [31].

All these results suggest that ANS measures derived from studies with different design characteristics cannot be compared directly, as the actual values of the respective measures not only depend on individual ANS acuity, but also on aspects of design characteristics. Design parameters can affect task performance by making the task more (or less) difficult or by inducing other solution strategies or additional processes. In case tasks differ only with respect to their difficulty, the actual values of the resulting ANS measures may be different, but they should be strongly related. However, if different aspects of task design induce differing solution strategies or cognitive processes, correlations between measures might be affected. Future research is needed to systematically evaluate the effects of different design characteristics on the resulting ANS measures. Moreover, we need a better understanding of the (additional) cognitive processes or strategies involved in the solution of the dot comparison task, as this also affects how the underlying ANS representations can be modelled.

Regarding comparability of the Weber fraction across studies it has to be considered that the values of the Weber fractions differ depending on whether they were fitted based on the linear or the logarithmic model. As can be seen from the descriptive statistics of Experiment 1, the Weber fractions resulting from the linear model are smaller than the Weber fractions from the logarithmic model. This further indicates that the results based on different studies and (moreover) different methods used to calculate the indices cannot be compared directly with regard to the overall size of the Weber fractions. Nevertheless, it is important to note that these differences in the overall size of the Weber fraction may not necessarily influence the correlations between the Weber fraction and other measures of ANS acuity or any external criterion variable (e.g., math achievement).

Future research is necessary to investigate whether the present findings can be generalized to other paradigms and age groups. Moreover, the effect of our manipulations on participants’ preferences regarding speed or accuracy was statistically significant, but nevertheless quite small. Varying not only task instructions or presentation durations but also the response window (i.e., the time window in which participants can indicate their response) might have a more pronounced impact on speed-accuracy trade-offs and, hence, on the results. This could be especially relevant for neuroimaging studies, as neuroimaging designs typically require limited response windows so that the haemodynamic response function can be detected best (cf. [108] for a discussion of the problem and possible solutions). Moreover, children at different ages might respond differently to design parameters influencing preferences for speed or accuracy, which might be important to consider when drawing conclusions regarding the development of ANS representations.

### Modeling ANS acuity–methodological aspects in calculating NRE_acc

NDE_acc and NRE_acc were often calculated analogously to NDE_RT and NRE_RT, for example by using a linear regression analysis with distance and ratio, respectively, as predictor [31]. Another often employed approach is categorizing the distances and ratios in small versus large distances and ratios, for example by defining the NRE_acc following [30]

$$NRE\_acc=\frac{mean\;accuracy\;for\;large\;ratios\;-mean\;accuracy\;for\;small\;ratios}{mean\;accuracy\;for\;large\;ratios}$$ (8)

(see e.g., [37,39]). All these procedures calculate NDE_acc and NRE_acc using a linear model, which may be problematic, as the linear model does not adequately fit the sigmoidal pattern of proportion data. Instead, the typical sigmoidal pattern for proportions, percentages or probabilities should be modeled using a generalized linear model using an appropriate link function. Relying on the wrong procedure, when calculating NDE_acc and NRE_acc, can have severe consequences for the relationship between these measures and other ANS measures, as we will show in the following simulation.

**Fig 11:**
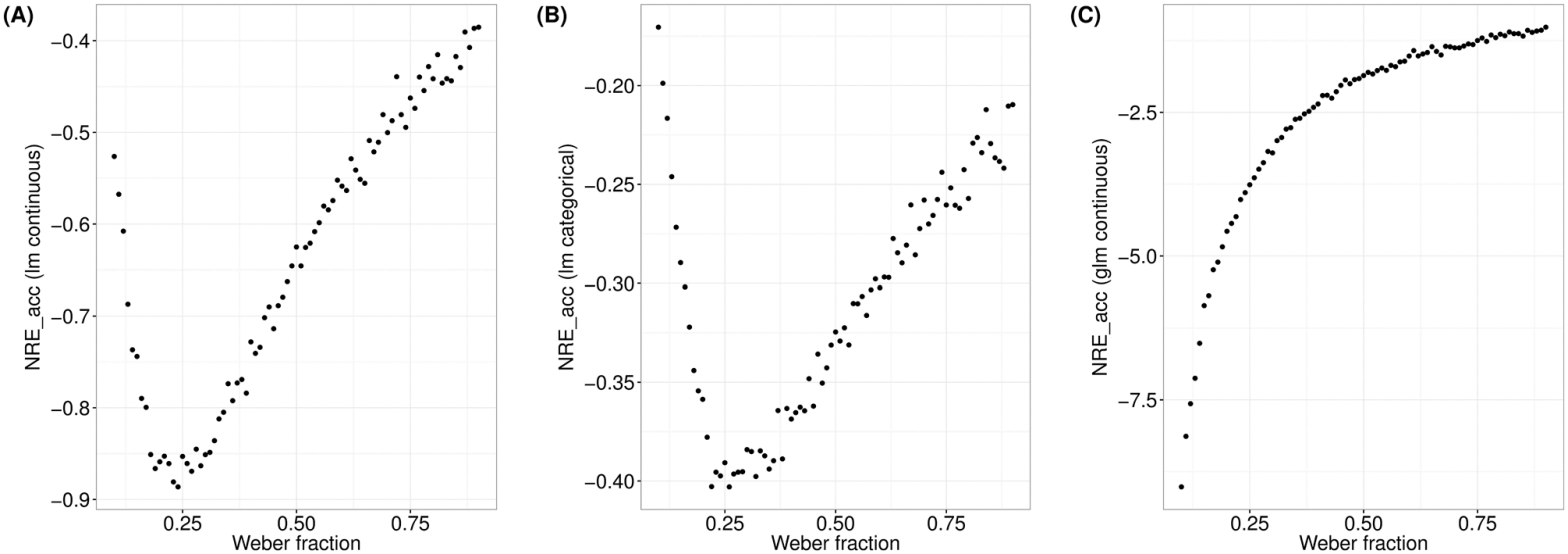
Relationship between NRE_acc and Weber fraction. NRE_acc was calculated (A) using a linear model with ratio as continuous predictor, (B) using a linear model with a categorical predictor, and (C) using a generalized linear model with ratio as continuous predictor.

We simulated accuracy data using the formula to estimate the Weber fraction according to the linear model (Eq 1) and a binomial sampling process (following [39]). In a first step, we calculated predicted accuracy (i.e., proportion correct) for each of the five ratios (i.e., 0.5, 0.6, 0.7, 0.8, and 0.9) for a given Weber fraction (see Eq 1). In total, we employed 81 Weber fractions ranging from 0.1 to 0.9 in steps of 0.01. Next, we simulated the responses of 100 participants per Weber fraction (i.e., a total of 8,100 participants) responding to 80 items per ratio (i.e. a total of 400 items per participant). To this end, responses were drawn from a binomial distribution using the predicted accuracy from step 1. Based on these simulated data we fitted the ratio effect for each participant separately using either a linear model or a generalized linear model employing the probit link function with ratio as predictor and accuracy as dependent variable. Then, we averaged the ratio effects calculated across Weber fractions. Fig 11 shows the mean simulated ratio effects as a function of the Weber fraction. We calculated NRE_acc (1) using a linear model with ratio as continuous predictor (panel A), (2) using a linear model with a categorical predictor (e.g., as in Eq 8, panel B), and (3) using a generalized linear model with binomial error distribution, probit as link function, and ratio as continuous predictor (panel C).

As can be seen in Fig 11, the relationship between Weber fraction and NRE_acc, when estimated using a linear model, is curvilinear. This property is problematic when using NRE_acc in diagnostic contexts, e.g. when testing for a potential deficit in ANS representations, because NRE_acc calculated from a linear model seems to be an ambiguous measure, which does not clearly indicate good or bad ANS acuity. The U-shaped relationship between the measures can also negatively affect results of standard correlation analyses, which cannot account for this U-shaped pattern. Depending on the distribution of the measures in the sample the resulting correlation between NRE_acc and other measures of ANS acuity (Weber fraction or mean accuracy) can either be high or low. Importantly, when calculating NRE_acc using a generalized linear model, the relationship between this measure and the Weber fraction is monotonous (see Fig 11) and, therefore, problems regarding the U-shaped relationship of these measures can be avoided. Thus, we recommend calculating the NRE_acc using a generalized linear model.

Finally, following these theoretical discussions we want to end this article with a discussion of the implications of our results for the repeatedly observed associations of ANS acuity and math performance [45,47–49] as a case in which above considerations and results may be meaningful.

### Implications for research on the relationship between ANS acuity and math performance

First of all, our experiments highlight the need for reliable measures, because unreliable measures attenuate the potentially observable size of a correlation [60]. Thus, using unreliable measures can result in artificially low correlations between ANS acuity and math performance, which might erroneously be interpreted as a lack of relationship between these two variables. The same argument applies to studies investigating differences between children with math learning difficulties and controls regarding their ANS acuity, because poor reliability makes it difficult to detect true differences between groups [109]. Neglecting the role of insufficient reliability can lead to wrong conclusions, as null effects might be misinterpreted as the absence of an effect instead of a lack of reliability [110].

Previous studies revealed that commonly used ANS measures differ regarding their reliability, whereby lower reliability was reported for NDE/ NRE than for mean accuracy or the Weber fraction (see [46] for a review). These differences might also contribute to the inconsistencies across existing studies addressing the relationship between ANS acuity and math performance, because the potentially observable size of the correlation is higher for measures with better reliability [46]. In line with this notion, a recent meta-analysis reported larger correlations between ANS acuity and math performance, when indexing ANS acuity using mean accuracy than when using the NDE [46,48]. So far, the majority of studies investigating the relationship between ANS acuity and math performance did not provide information regarding the reliability of the measures employed. For example, only 10 out of 36 studies in the meta-analysis of Chen and Li (2014) reported the reliability of the ANS measures. Moreover, only few studies used more than 400 trials [42,92,111], which were suggested to be necessary to reach acceptable reliability [39]. Given the importance of reliability, it is crucial to check the actual reliability of the measures and to aim at sufficient reliability by employing a large number of trials. The present experiments show that also the NDE/ NRE can constitute reliable measures, when acting accordingly.

More importantly, however, the main finding of the present experiments is that RT based measures do not assess ANS acuity in the same way as accuracy based measures. This result suggests that findings from studies using RT based measures cannot be compared directly with results from studies using accuracy based measures, because these types of measures do not correspond sufficiently in assessing ANS acuity. Moreover, the results of Experiments 2 and 3 indicate that accuracy may be more informative about the underlying ANS representations than RT. These differences between accuracy and RT based measures may also explain the so far inconsistent results regarding correlations between ANS acuity and math performance. When accuracy based measures are indeed more informative about ANS acuity, math performance should be correlated more strongly with accuracy based measures than with RT based measures. In line with this conclusion, Fazio and colleagues [49] found somewhat larger correlations between ANS acuity and math performance for accuracy based measures (acc: *r* = .29; w: *r* = .19) than for RT based measures (mean RT: *r* = .09).

Moreover, our simulation revealed that NRE_acc when based on a linear model is related to ANS acuity indexed by the Weber fraction in a curvilinear manner. Thus, these measures can be related positively or negatively with ANS acuity depending on the distribution of ANS acuity (i.e., Weber fractions) in the respective sample–which can result in either positive, negative, or even null correlations with math performance (see also [112], for a discussion on curvilinear relationships between distance effect and math performance for symbolic number comparison depending on individual math capability). This might also account at least in part for the so far inconsistent results regarding correlations between NRE_acc and NDE_acc and mathematical abilities [30,37–39,113]. This problem can be avoided in future research when (1) calculating the NRE_acc or the NDE_acc using a generalized linear model with the probit as link function, because in this case the relationship with ANS acuity indexed by the Weber fraction is monotonous, or (2) relying on mean accuracy or the Weber fraction.

## Conclusions

Taken together, our results demonstrated that–given sufficient reliability–all accuracy based measures (i.e., mean accuracy, Weber fraction, NDE_acc, and NRE_acc) were highly correlated, as expected based on the mathematical relations between distance, ratio and the Weber fraction. This suggests that all these measures can be used interchangeably to assess ANS acuity. Regarding RT based measures (i.e., mean RT, NDE_RT, and NRE_RT) our results consistently revealed a high correlation between NDE_RT and NRE_RT, whereas the correlations between these two measures and mean RT were substantially smaller and depended on the presentation duration of the stimuli. Thus, only NDE_RT and NRE_RT may be used interchangeably. Moreover, our results revealed that the inverse efficiency score correlates primarily with mean RT. Thus, an alternative composite score (the *z*-score) was proposed capturing variance of accuracy and RT in a more balanced way. This measure can also be used interchangeably with accuracy based measures (or mean RT) and might also constitute a measure controlling for a speed-accuracy trade-off.

Most important from a theoretical point of view, however, was our finding that RT and accuracy based measures do not correspond in assessing ANS acuity. In particular, our results revealed a speed-accuracy trade-off with the consequence that accuracy and RT based measures provided opposing conclusions about ANS acuity. Therefore, these types of measures cannot be used interchangeably. These differences between accuracy and RT based measures may also explain the so far inconsistent results regarding the relationship between ANS acuity and math performance.

From an applied perspective, our findings raised the question which measure should be preferred as an index of ANS acuity. The present results indicated that accuracy based measures were especially informative about the underlying ANS representations. Thus, it may generally be recommended to use accuracy based measures to assess ANS acuity. More specifically, our data indicated that using instructions emphasizing accuracy (over speed) and avoiding too short presentation durations of the stimuli (like 50-200ms) may be beneficial.

## Supporting Information

S1 File Demographic data. (XLSX)

S2 File Experimental data. (XLSX)
